# Re-establishment of H3K9me2 eliminates the transcriptional inhibition of ST18 on meiotic genes and orchestrates female germ cell development

**DOI:** 10.1093/nar/gkaf657

**Published:** 2025-07-12

**Authors:** Bing-Wang Zhao, Yi-Na Zhang, Tie-Gang Meng, Yuan-Hong Xu, Yi-Ke Lu, Si-Min Sun, Jia-Ni Guo, Xue-Mei Yang, Zhen-Bo Wang

**Affiliations:** Key Laboratory of Organ Regeneration and Reconstruction, Institute of Zoology, Chinese Academy of Sciences, Beijing 100101, China; Institute for Stem Cell and Regeneration, Chinese Academy of Sciences, Beijing 100101, China; Beijing Institute for Stem Cell and Regenerative Medicine, Beijing 100101, China; University of Chinese Academy of Sciences, Beijing 100049, China; Key Laboratory of Organ Regeneration and Reconstruction, Institute of Zoology, Chinese Academy of Sciences, Beijing 100101, China; Institute for Stem Cell and Regeneration, Chinese Academy of Sciences, Beijing 100101, China; Beijing Institute for Stem Cell and Regenerative Medicine, Beijing 100101, China; University of Chinese Academy of Sciences, Beijing 100049, China; Guangzhou Key Laboratory of Metabolic Diseases and Reproductive Health, Guangdong-Hong Kong Metabolism & Reproduction Joint Laboratory, Reproductive Medicine Center, the Affiliated Guangdong Second Provincial General Hospital of Jinan University, Guangzhou, 510317, China; Key Laboratory of Organ Regeneration and Reconstruction, Institute of Zoology, Chinese Academy of Sciences, Beijing 100101, China; Institute for Stem Cell and Regeneration, Chinese Academy of Sciences, Beijing 100101, China; Beijing Institute for Stem Cell and Regenerative Medicine, Beijing 100101, China; University of Chinese Academy of Sciences, Beijing 100049, China; Key Laboratory of Organ Regeneration and Reconstruction, Institute of Zoology, Chinese Academy of Sciences, Beijing 100101, China; Institute for Stem Cell and Regeneration, Chinese Academy of Sciences, Beijing 100101, China; Beijing Institute for Stem Cell and Regenerative Medicine, Beijing 100101, China; University of Chinese Academy of Sciences, Beijing 100049, China; Key Laboratory of Organ Regeneration and Reconstruction, Institute of Zoology, Chinese Academy of Sciences, Beijing 100101, China; Institute for Stem Cell and Regeneration, Chinese Academy of Sciences, Beijing 100101, China; Beijing Institute for Stem Cell and Regenerative Medicine, Beijing 100101, China; University of Chinese Academy of Sciences, Beijing 100049, China; Key Laboratory of Organ Regeneration and Reconstruction, Institute of Zoology, Chinese Academy of Sciences, Beijing 100101, China; Institute for Stem Cell and Regeneration, Chinese Academy of Sciences, Beijing 100101, China; Beijing Institute for Stem Cell and Regenerative Medicine, Beijing 100101, China; University of Chinese Academy of Sciences, Beijing 100049, China; Key Laboratory of Organ Regeneration and Reconstruction, Institute of Zoology, Chinese Academy of Sciences, Beijing 100101, China; Institute for Stem Cell and Regeneration, Chinese Academy of Sciences, Beijing 100101, China; Beijing Institute for Stem Cell and Regenerative Medicine, Beijing 100101, China; University of Chinese Academy of Sciences, Beijing 100049, China; Key Laboratory of Organ Regeneration and Reconstruction, Institute of Zoology, Chinese Academy of Sciences, Beijing 100101, China; Institute for Stem Cell and Regeneration, Chinese Academy of Sciences, Beijing 100101, China; Beijing Institute for Stem Cell and Regenerative Medicine, Beijing 100101, China; University of Chinese Academy of Sciences, Beijing 100049, China

## Abstract

Epigenetic reprogramming is widespread and highly active during gametogenesis, which is usually involving in the expression of critical genes. The expression of genes couple with transcription activation, and the transcriptional regulation by transcription factors predetermine protein translation for biological processes. In this study, we found that EHMT1-mediated re-establishment of H3K9me2 played crucial roles in the progression of meiosis in female germ cells. EHMT1 deficient female mice were nearly infertile due to the arrest of zygotene in embryonic germ cells, which was caused by downregulated expression of key meiotic genes. Furthermore, we identified transcription suppressor, particularly ST18, for meiotic genes by combining RNA-seq, Cut&Tag seq analysis, and luciferase reporter assays. We uncovered that H3K9me2 mediated ST18 expression homeostasis and played critical roles in regulating the timed expression of key meiotic genes. Overall, we revealed that EHMT1-mediated H3K9me2 re-establishment facilitated the expression of key meiotic genes for female meiosis progression.

## Graphical Abstract

**Graphical Abstract:**
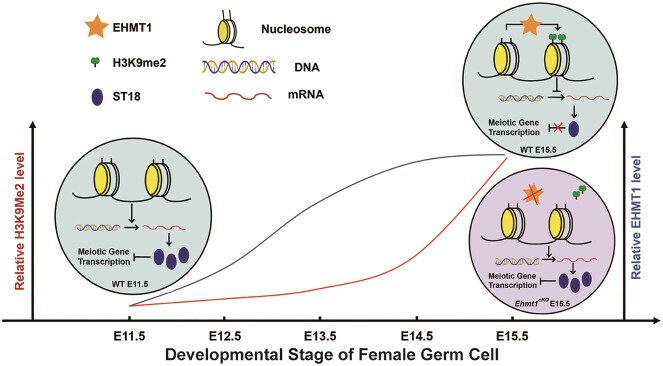

## Introduction

Differentiated cells typically maintain stable gene expression through epigenetic mechanisms including DNA methylation and covalent modifications [1, 2]. The germ cell genome, as the source for the next generation, must maintain an epigenetic "reprogrammable" state to facilitate the creation of new generations. In mice, primordial germ cells (PGCs) initiate DNA demethylation around E7.25, reaching a minimum level by E13.5. In female germ cells, this low level of DNA methylation persists until after birth [2]. Previous studies by Saitou group found that PGCs experienced significant loss of H3K9me2 (mediated by downregulation of EHMT1) at E8.0 prior to their colonization, while high levels of H3K27me3 were acquired at E9.0 [3, 4]. This suggests that such reprogramming is crucial for the potential pluripotency of PGCs. After colonization, the PGCs immediately undergo sexual differentiation [5], and those PGCs that differentiate into female germ cell quickly enter meiosis [6, 7]. However, the changes in histone modifications of oogonia after colonization are unknown.

Meiosis is a hallmark of gametogenesis in sexually reproducing organisms, ensuring genetic diversity through recombination [8]. The activation of the key regulatory factor STRA8 (stimulated by retinoic acid) initiates the entry of germ cells into meiosis [9]. Programmed DNA double-strand breaks (DSBs) occur during the leptotene stage of prophase I. During zygotene and pachytene, these DSBs are gradually repaired by homologous recombination (HR), promoting and completing homologous pairing [10]. DSBs generated by SPO11 initiates meiotic recombination. Replication protein A (RPA) coats single-stranded DNA (ssDNA), forming nucleoprotein filaments that prevent degradation of ssDNA and secondary structure formation [11]. The recombinases RAD51 and DMC1 cover the 3′ single strand and facilitate strand invasion of homologous chromosomes [12, 13]. The interaction between RPA and MEIOB-SPATA22 mediates a second end capture, leading to the displacement of one strand of the homolog and D-loop formation [8]. The other end of the DSBs is "captured" , resulting in the formation of a double Holliday junction stabilized by MSH4 and MSH5 to promote crossing over [14]. Proteins such as SYCP1, SYCP3, SYCE1, SYCE3, and TEX1 are involved in the assembly of the synaptonemal complex [15, 16].

Histone methylation is crucial for the regulation of chromatin structure and gene expression, and is widely involved in the development and maturation of germ cells [17]. Many histone methyltransferases have been reported to participate in meiosis by regulating histone modifications and gene expression [18]. The histone methyltransferase PRDM9 could participate in the initiation of DSBs in female germ cells by promoting various types of histone H3 modifications (H3K4me3, H3K9me2/3, and H3K36me3) [19]. The H3K9 methyltransferase EHMT2 (G9a) plays a pivotal role in HR during female germ cell meiosis and depletion of EHMT2 disrupts the completion of DSBs repair, ultimately leading to female sterility [20]. More studies have reported the functions of histone methyltransferases in spermatogenesis, mainly because male germ cells are more readily accessible for the study of meiotic processes. It is worthy to note that meiotic processes in germ cells appear to differ, for instance, H3K9me2 modifications persist during prophase I in females but are present only before pachytene in male germ cells [20].

EHMT1, a histone methyltransferase, is primarily localized in euchromatin and catalyzes the mono- and di-methylation of histone H3 at lysine 9 (H3K9me1/2) [21], and its functions are similar to those of EHMT2 and contribute to transcriptional silencing [22–24]. EHMT1 and EHMT2 typically function as a heterodimer to exert their catalytic activity [21], due to knockout (KO) mice for either gene display similar phenotypes, including the loss of H3K9me2 modifications prior to birth and embryonic lethality [25]. Although EHMT1 and EHMT2 are generally considered functionally interdependent, increasing evidence in recent years has revealed EHMT1-specific roles independent of EHMT2, particularly during oocyte maturation [26] and embryonic development [27]. EHMT1 participates in localized transcriptional repression during the transition of oocytes from the NSN stage to the SN stage, partially independent of EHMT2 [26]. Additionally, EHMT1 has been reported to play a role in the establishment of H3K27me2 in the paternal pronucleus [27].

In this study, we found that female germ cells underwent dramatic epigenetic reprogramming after colonizing the gonadal ridges, which was primarily characterized by the reconstruction of H3K9me2. This reprogramming was mainly mediated by the histone methyltransferase EHMT1. Additionally, we discovered that *Ehmt1*-conditional knockout (cKO) mice lost fertility due to the arrest of meiotic prophase I. RNA-seq analysis revealed that the absence of EHMT1 led to downregulation of genes essential for meiosis. Furthermore, combined with Cleavage Under Targets & Tagmentation followed by sequencing (CUT&Tag sequencing) analysis of H3K9me2, we observed that the expression of two transcription suppressors, ST18 [28] and ZFP462 [29], increased after the depletion of H3K9me2. The upregulation of these two severely inhibited the transcription of critical meiotic genes, resulting in zygotene arrest. Remarkably, we found that ST18 remains highly expressed in female germ cells during mitosis (E11.5). Given the abnormal upregulation of ST18 in Ehmt1-deficient germ cells during meiosis (E15.5), we infer that the high expression of ST18 inhibits the transcription of meiotic genes during germ cell mitosis, thereby controlling the entry of meiosis on time. Our findings revealed that the homeostasis of transcriptional repressors mediated by H3K9me2, a histone modification mediated by EHMT1, plays a crucial role in regulating the transcription of key meiotic genes and orchestrating female germ cell meiosis progression.

## Materials and methods

### Animals

Mice lacking *Ehmt1* in PGCs (referred to as *Ehmt1^cKO^*) were generated by crossing *Ehmt1^F/F^* mice with transgenic mice expressing *Tnap* promoter mediated Cre recombinase. All mice used in this work had a C57BL/6J genetic background. All mice used in this study were maintained in Laboratory Animal Center, Institute of Zoology, Chinese Academy of Sciences. Ambient temperature was 21°C–25°C, relative humidity was 40%-60%, and lighting was provided on a 12-h alternating light/dark cycles. All of the animal experiments were performed according to the Animal Research Committee principles of the Institute of Zoology, Chinese Academy of Sciences.

### Fertility assessment of *Ehmt1^cKO^* mice

The fertility assessment experiments were performed as follow: Control and *Ehmt1^cKO^* female mice at sexual maturity were continually mated to wild-type male mice for 6 months. At least five mice of each genotype were used. Cages were checked daily for counting the number of litters and pups.

### Immunoblotting

Proteins from cells or tissues were extracted in Radio Immunoprecipitation Assay (RIPA) lysis buffer with a protease inhibitor mixture (Roche). After grinding of ovary in a homogenizer, the samples were incubated on ice for 30 min. Next, the protein lysates were transferred to pre-cooled EP tubes and centrifuged at 13 500 × *g* for 15 min at 4°C. The supernatants of the extracts were collected and boiled at 95°C for 5 min, then immunoblotting was performed. In brief, the boiled proteins were separated in sodium dodecyl sulfate–polyacrylamide gel electrophoresis, then transferred to polyvinylidene fluoride membrane. After incubated with 5% bovine serum albumin (BSA; room temperature for 1–2 h), primary antibodies (4°C for overnight) and secondary antibodies (37°C for 1 h), the membrane was scanned with the enhanced chemiluminescence detection system (Bio-Rad). The antibodies used are provided in the Supplementary Table S2.

### Immunofluorescence staining

Immunofluorescence staining of ovarian sections was performed as follows: Ovaries were fixed in 4% paraformaldehyde, dehydrated, and embedded in paraffin. Then ovaries were cut into sections of 5 μm thickness. Then, the sections were deparaffinized, immersed in antigen retrieval solution (sodium citrate buffer), and heated for 15 min. After blocked with 5% BSA, sections were incubated with primary antibodies and appropriate fluorescein isothiocyanate-conjugated secondary antibodies. Last, the nuclei were stained with 4′,6-diamidino-2-pheny-lindole (DAPI). Images were captured using a laser scanning confocal microscope (Leica Stellaris).

Embryonic germ cell surface spreading was referred to a method previously described with a slight modification [30]. Briefly, the ovaries were washed in phosphate-buffered saline (pH = 7.4) and then placed in a hypotonic extraction buffer containing 30 mM Tris, 50 mM sucrose, 17 mM trisodium citrate dihydrate, 5 mM ethylenediaminetetraacetic acid (EDTA), 0.5 mM Dithiothreitol (DTT), and protease inhibitor (Roche) for 1.5 h. Then the ovaries were transferred to a 200 mM sucrose solution, shredded, and pipetted repeatedly to make suspensions. The cell suspensions were placed on slides containing 1% paraformaldehyde and 0.15% Triton X-100. The slides were dried for overnight in a closed box with high humidity. After incubated with 5% BSA, primary antibodies, secondary antibodies, and DAPI, the dried slides were scanned with the laser scanning confocal microscope (Leica Stellaris).

The antibodies used are provided in the Supplementary Table S2.

### Immunofluorescence quantification method

For quantification of immunofluorescence signal intensity, cell samples from different time points or genotypes were imaged under identical acquisition settings. The resulting immunofluorescence images were then processed using ImageJ software to measure signal intensity within target cells or regions of interest. Additionally, for standardization with somatic cells as the reference, we first collected and averaged the fluorescence signal density of somatic cells. Then, the fluorescence signal density of germ cells was normalized by dividing it by the average fluorescence signal density of somatic cells. This allowed for consistent and quantitative comparison of fluorescence signal levels.

### RNA extraction and complementary DNA library preparation

Total RNAs were extracted using the TRIzol reagent (Tiangen Biotech; China; DP424). Reverse Transcription Reagent Kit (abm, Vancouver, BC, Canada) was used to establish a complementary DNA library with a polymerase chain reaction (PCR) instrument. Real-time polymerase chain reaction (RT-PCR) analysis was conducted using SYBR^®^ for quantification. The sequences of RT-PCR primers are provided in the Supplementary Table S1.

### TUNEL analysis

For slides, apoptotic cells were detected using the One Step method, following the manufacturer’s instructions for the TUNEL Apoptosis Detection Kit (Meilunbio, MA0223) for slide analysis. The dried slides were scanned with the laser scanning confocal microscope (Leica Stellaris).

For ovarian cells, apoptotic cells were detected using TUNEL BrightGreen Apoptosis Detection Kit (Vazyme Biotech Co., Ltd, A112) for cells analysis. The slides with cells were scanned with the laser scanning confocal microscope (Leica Stellaris).

### Cell culture and transfection

HEK293T cells were cultured in Dulbecco’s modified Eagle’s medium supplemented with 10% Fetal Bovine Serum (FBS) at 37°C in a 5% CO_2_ incubator. Transfections of plasmids were performed using Lipofectamine 3000 (Thermo Fisher), according to the manufacturer’s instructions. Cells were harvested 48 h post-transfection.

### Plasmid construction and luciferase reporter assay

The full-length CDS of *St18, Zfp462, Etv6*, and *Klf12* were cloned respectively into a vector containing the CMV promoter and *Myc* tag. PCR amplification of the transcription start sites (TSS) (∼2500–3000 bp) of meiotic genes *Mei1, Dmc1, Meiob, Spata22, Msh4*, and *Syce3* was performed respectively, and the amplified fragments were ligated into the pRP-hRluc/Puro-miniCMV-luciferase (Vectorbuilder) vector. HEK293T cells were co-transfected with luciferase reporter plasmids and overexpression plasmids. After 48 h culture, cells were harvested and lysed. Luciferase activity was measured using a dual-luciferase reporter assay system, following the manufacturer’s instructions (Vazyme Biotech Co., Ltd, DL101). Primer sequences for plasmid construction are listed in the Supplementary Table S1.

### RNA-seq and data analysis

E15.5 female germ cells with Tg (*Oct4*-eGFP) were obtained using fluorescence-activated cell sorting (FACS). In short, the embryonic ovaries were digested into a single-cell suspension, and *Oct4*-eGFP positive cells were collected using LSRFortessa X-20 (BD Biosciences) or FACS Fusion (BD Biosciences). Each group contained about 3000 female germ cells, and three replicates were prepared to RNA sequencing for both Control and *Ehmt1^cKO^* groups. Raw RNA-seq reads were processed for quality assessment using FastQC (v0.12.1) and trimmed to remove adapters and low-quality bases with Trim Galore (v0.6.10) under default parameters. HISAT2 (v2.21) was used to map the trimmed reads to the mouse reference genome (GRCm39), which was downloaded from the ENCODE database, using default parameters. Stringtie (v2.2.1) was used for gene expression quantification, and the resulting transcript quantification files were merged into a unified gene expression matrix using the prepDE.py script. Differential expression analysis was conducted using DESeq2 (v1.38.3). This tool models normalized read counts with a negative binomial distribution, estimates gene-wise variance across samples, and calculates adjusted p-values (padj) to identify significantly differentially expressed genes (DEGs). DEGs in DESeq2 analysis were defined as fold-change > 2.0 and padj < 0.05. Gene ontology (GO) analysis was performed using the DAVID database.

### Purification of germ cells by MACS

Isolation of female germ cells was performed as reported in the previous work [31]. Embryonic gonads were isolated, and the mesonephros was removed. The gonads were then digested into a single-cell suspension using a digestion buffer containing 0.25% trypsin and 1 mM EDTA. After washing with Dulbecco's Phosphate Buffered Saline (DPBS buffer) (with 0.5% BSA and 1 mM EDTA, without calcium and magnesium ions), the suspension was incubated with SSEA1 antibody-coated beads (Miltenyi Biotec, cat. no. 130-094-530) for 10 min. Magnetic separation was employed to isolate SSEA1 antibody-labeled germ cells from unlabeled somatic cells, resulting in highly purified germ cells.

### CUT&Tag assay and analysis

CUT&Tag sequencing was performed according to the Hyperactive Universal CUT&Tag Assay Kit (Vazyme, Nanjing, China, TD904). Paired-end reads were trimmed using Trim Galore (v0.6.10) with default parameters. The trimmed reads were aligned to the mouse reference genome (GRCm39) using Bowtie2 (v2.5.3) with the following parameters: –end-to-end, –no-mixed, –no-discordant. SAM files were converted to BAM files, and unmapped reads as well as reads with an alignment quality score below 10 were filtered out. PCR duplicates were removed using Picard MarkDuplicates (v3.1.1). For peak calling, MACS2 (v2.2.9.1) was used with the parameters –broad and -p 0.01. Differential peak analysis was performed using the DiffBind package (v3.8.4) with a significance threshold of fold-change > 2 and *P*-value < 0.05. Peaks were annotated using the ChIPseeker package (v1.34.1) in combination with the mouse genome annotation database (TxDb.Mmusculus. UCSC. mm39. refGene). To assess group similarity, BAM files were processed using bamCoverage to generate normalized BigWig files. The similarity between groups was then evaluated using multiBigwigSummary followed by plotCorrelation from deepTools (v3.5.5). Differentially modified regions were used to generate average signal profiles for H3K9me2 between the control and experimental groups using the computeMatrix command. The resulting data were further visualized using ggplot2. GO analysis was performed using the DAVID database.

For overall H3K9me2 signal intensity analysis. Signal intensities were normalized using log_2_(experimental signal/background signal) to enable accurate comparison of histone modification levels.

### Statistical analysis

The Photoshop CC 2015 (Adobe) and Illustrator CC 2015 (Adobe) were used for analysis of the images, including image layout, highlighting key cells, annotating marker proteins, and image cropping. Quantitative analysis of each experiment (mean ± standard error of mean) was obtained from repeating at least three times and processed by Student’s *t*-test using Prism8 (GraphPad Software). A value of *P* < 0.05 was considered statistically significant for any differences. *P*-values are denoted in figures or figure legends. *, **, and *** represent *P*< 0.05, *P*< 0.01, and *P*< 0.001, respectively.

## Results

### Dynamics of histone modifications in female germ cells after colonization

**Figure 1.**
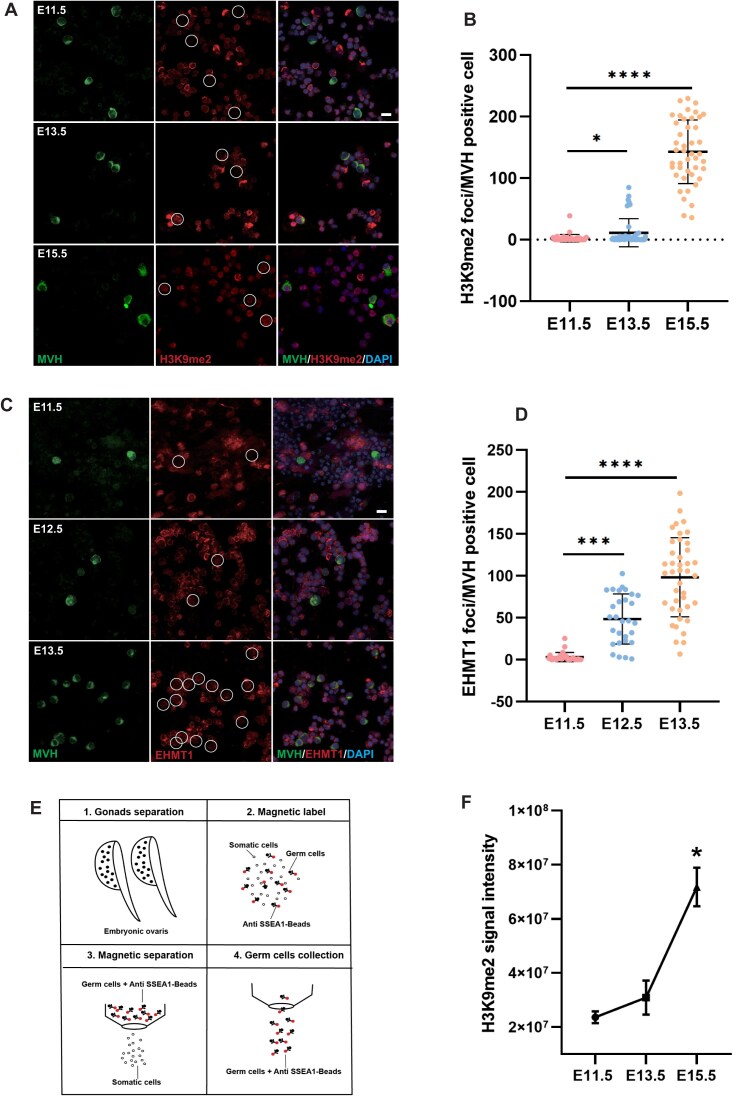
Dynamics of histone modifications and EHMT1 in female germ cells. (**A**, **B**) Immunofluorescence staining of ovarian cells at E11.5, E13.5, and E15.5 using anti-H3K9me2, anti-MVH, and DAPI. Dashed circles represent germ cells (**A**), and fluorescence quantification for H3K9me2 in MVH-positive cells (**B**). The data of quantitative analysis were obtained from 20 replicates. Scale bar = 10 μm. Data are expressed as mean ± standard deviation (SD). Two-tailed Student’s *t*-test; *P* < 0.05 (*), *P* < 0.00001 (****). (**C**, **D**) Immunofluorescence staining of ovarian cells at E11.5, E12.5, and E13.5 using anti-EHMT1, anti-MVH, and DAPI. Dashed circles represent germ cells (**C**), and fluorescence quantification for EHMT1 in MVH-positive cells (**D**). The data of quantitative analysis were obtained from 20 replicates. Scale bar = 10 μm. Two-tailed Student’s *t*-test; *P* < 0.001 (***), *P* < 0.01 (****). (**E**) Experimental workflow for collecting SSEA1 positive cells: Ovaries from E15.5 embryos are isolated (**1**), single-cell suspensions are prepared and incubated with Anti-SSEA1 beads (**2**), SSEA1-positive cells are magnetically isolated (**3**), and SSEA1-positive cells are collected (**4**). (**F**) Overall H3K9me2 signal intensity curve for E11.5, E13.5, and E15.5 stages.

Before colonizing the genital ridge, PGCs undergo extensive epigenetic reprogramming, characterized by the loss of DNA methylation [2] and H3K9me2, as well as the acquisition of high levels of H3K27me3 [4]. Sexual differentiation begins after PGCs colonization, thereafter the female PGCs develop into oogonia [32]. Therefore, we focused on how these epigenetic modifications change in oogonia during their mitosis and meiosis after colonizing the genital ridge. We measured the epigenetic modifications of oogonia at different developmental stages (E11.5, E13.5, E15.5) by immunofluorescence staining and counting statistics. As shown in Fig. 1A and B, the level of H3K9me2 in oogonia is very low before E13.5 but increases markedly by E15.5. In contrast, the level of H3K27me3 changes only slightly during the E11.5–E15.5 period (Supplementary Fig. S1A and B). To further substantiate the reprogramming of H3K9me2 in female germ cells, we isolated germ cells using anti-SSEA-1 magnetic beads and performed Cut&Tag sequencing, a highly efficient chromatin protein modification analysis method based on Tn5 transposase technology. As shown in Fig. 1E, we first isolated the gonads, then digested them into a single-cell suspension. And the cell suspension was then incubated with SSEA1 antibody magnetic beads, and unconjugated cells were removed using magnetic separation, with the germ cells being collected at the end. To verify whether this method is applicable for collection of female germ cells from E15.5 embryo, we performed immunofluorescence staining of SSEA1 positive cells and MVH was used as a germ cells maker. We found that the rate of collected MVH positive cells was > 90% (Supplementary Fig. S1E). The quantitative analysis of H3K9me2 signal density confirmed that H3K9me2 was re-established during the developmental stages from E11.5 to E15.5 (Fig. 1F).

Previous work confirmed that EHMT1, as a histone methyltransferase, plays an important role in the conferment of H3K9me2 and H3K27me3 [4, 27]. Then we examined the dynamic changes of EHMT1 in this process. Interestingly, EHMT1 was expressed in low abundance at E11.5 but was rapidly increased and detected in most oogonia by E13.5 (Fig. 1C and D). It was obvious that the expression of EHMT1 precedes the re-establishment of H3K9me2, both showing simultaneous accumulation over time. Moreover, given that EHMT1 is the methyltransferase responsible for H3K9me1/2 and shows progressive accumulation during E11.5–E13.5, we sought to examine the dynamics of H3K9me1. We observed that H3K9me1 remained at low levels in female germ cells throughout E11.5–E15.5, with no discernible temporal changes (Supplementary Fig. S1C and D). Based on these observations, we hypothesized that EHMT1 may be essential for the re-establishment of H3K9me2 during this process. We then analyzed the function of EHMT1 in oogonia development.

### EHMT1 is essential for female mice fertility

**Figure 2.**
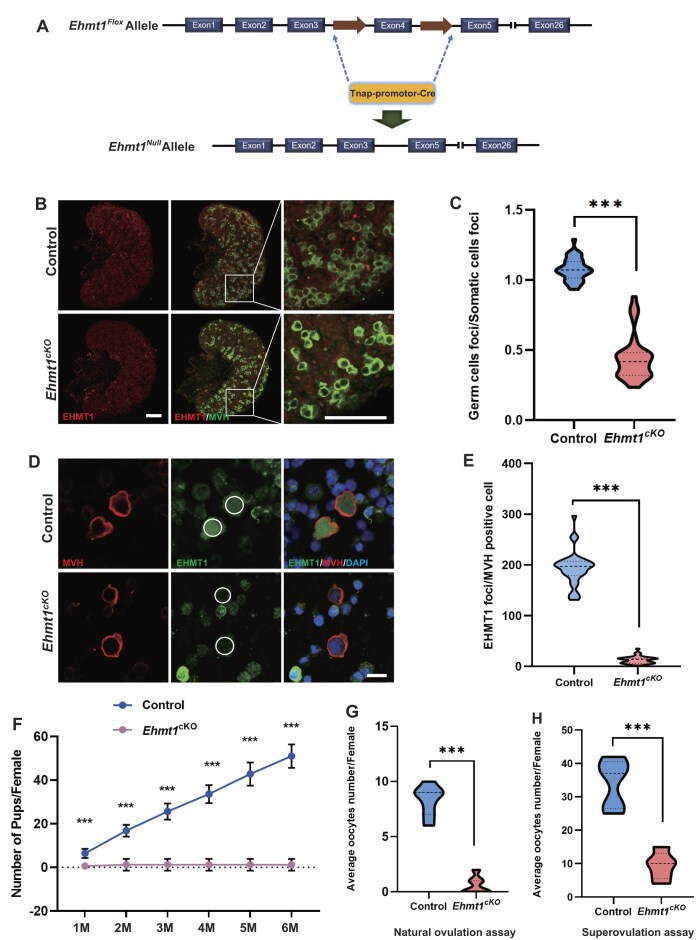
EHMT1 depletion causes female sterility. (**A**) Schematic of the germ cell specific *Ehmt1* exon4 deletion mediated by *Tanp*-Cre recombinase. (**B**, **C**) Immunofluorescence staining of EHMT1 in ovarian sections of Control and *Ehmt1^cKO^* embryo, MVH co-staining indicates the position of germ cells. Enlarged images are shown in the right panel, DNA is stained with DAPI. Scale bar = 25 μm (**B**), and quantification of EHMT1 foci of germ cells/somatic cells from Control and *Ehmt1^cKO^* embryos (**C**). Embryos were obtained from the same pregnant female and analyzed at E16.5. The data of quantitative analysis were obtained from 40 replicates of 3 embryos. Data are expressed as mean ± SD. Two-tailed Student’s *t*-test; *P* < 0.001 (***). (**D**, **E**) Immunofluorescence staining of EHMT1 in digested ovarian cells of Control and *Ehmt1^cKO^* embryos, MVH co-staining indicates the position of germ cells. DNA is stained with DAPI. Scale bar = 10 μm (**D**), and fluorescence quantification for EHMT1 in MVH-positive cells from Control and *Ehmt1^cKO^* embryos (**E**). Embryos were obtained from the same pregnant female and analyzed at E13.5. The data of quantitative analysis were obtained from 40 replicates of 3 embryos. Data are expressed as mean ± SD. Two-tailed Student’s *t*-test; *P* < 0.001 (***). (**F**) Breeding assays of Control (n = 5) and *Ehmt1^cKO^* (n = 5) female mice. Continuous breeding assessment showed the cumulative number of pups per Control and *Ehmt1^cKO^* female mouse for 6 months. Data are expressed as mean ± SD. Two-tailed Student’s *t*-test; *P* < 0.01 (**), *P* < 0.001 (***), *P* < 0.00001 (****). (**G**) Natural ovulation assay in Control (n = 7) and *Ehmt1^cKO^* (n = 7) female mice. Data are expressed as mean ± SD. Two-tailed Student’s *t*-test; *P* < 0.001 (***). (**H**) Superovulation assay in Control (n = 5) and *Ehmt1^cKO^* (n = 5) female mice. Data are expressed as mean ± SD. Two-tailed Student’s *t*-test; *P* < 0.001 (***).

To explore the function of EHMT1 in oogenesis and female fertility, we mated *Ehmt1^Flox/Flox^* (*Ehmt1^F/F^*) mice, in which the exon 4 was flanked by two loxP sites, with *Tnap*-Cre transgenic mice in which the Cre recombinase driven by a *Tnap* promoter is specifically expressed in PGCs (Fig. 2A) [33]. *Ehmt1^F/+^* -*Tnap*-Cre males and *Ehmt1^F/F^* females were used for breeding, and their offspring were utilized for subsequent experimental analyses. Four genotypes of mice were obtained, including *Ehmt1^F/+^*, *Ehmt1^F/−^*, *Ehmt1^F/+^*-*Tnap*-Cre, and *Ehmt1^F/−^*-*Tnap*-Cre. We used *Ehmt1^F/+^* mice (noted as Control) and *Ehmt1^F/−^*-*Tnap*-Cre mice (noted as *Ehmt1^cKO^*) in the following experiments. We performed immunoblotting analysis on embryonic ovaries of E13.5 females, and observed that EHMT1 was slightly reduced in *Ehmt1^cKO^* ovaries compared with Control group (Supplementary Fig. S2B and C). To avoid interference of somatic cells, we next performed RT-PCR and immunofluorescence staining. RT-PCR analysis on sorted germ cells validated that *Ehmt1* mRNA were depleted in *Ehmt1^cKO^* mice, showing near-undetectable levels versus Control (Supplementary Fig. S2A). And the loss of EHMT1 in *Ehmt1^cKO^* female germ cells was more intuitively demonstrated by immunofluorescence analysis of ovaries sections (Fig. 2B and C). Similarly, immunocytochemical analysis of oogonia isolated from ovaries also confirmed that EHMT1 was absent in female germ cells of *Ehmt1^cKO^* ovaries (Fig. 2D and E).

After 6 months of breeding assays, we found that the *Ehmt1^cKO^* female mice were almost infertile (Fig. 2F). Both natural ovulation assays and superovulation assays confirmed that the *Ehmt1^cKO^* female mice could only ovulate very few oocytes (Fig. 2G and H).

### EHMT1 deletion results in severe loss of germ cells

**Figure 3.**
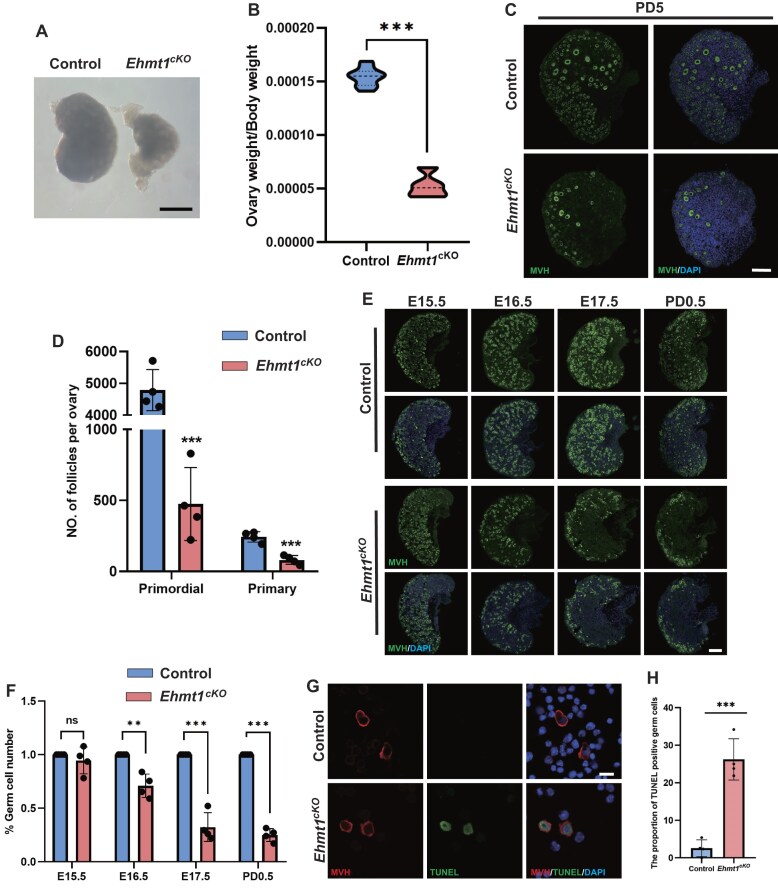
Apoptosis of female germ cells in *Ehmt1^cKO^* embryos at E16.5. (**A**) Comparison of morphological features between Control (n = 8) and *Ehmt1^cKO^* (n = 8) ovaries. Mice were born in the same litters and analyzed at PD20. Scale bar = 1 mm. (**B**) Ovary weight/body weight ratio of Control (n = 4) and *Ehmt1^cKO^* females (n = 4), Data are expressed as mean ± SD. Two-tailed Student’s *t*-test; *P* < 0.001(***). (**C** and **D**) Immunofluorescence staining of MVH in Control and *Ehmt1^cKO^* ovaries, with DNA labeled by DAPI. Scale bar = 20 μm (**C**). Follicle counts in Control (n = 4) and *Ehmt1^cKO^* (n = 4) ovaries at PD0.5 (**D**). Embryos were born in the same litters and analyzed at PD0.5. Primordial: Primordial Follicles, Primary: Primary Follicles, Data are expressed as mean ± SD. Two-tailed Student’s *t*-test; *P*< 0.001 (***). (**E**, **F**) Immunofluorescence staining of MVH in Control and *Ehmt1^cKO^* ovaries, with DNA labeled by DAPI. Scale bar = 25 μm (**E**). Germ cells number quantification of Control (n = 4) and *Ehmt1^cKO^* (n = 4) ovaries at E15.5, E16.5, E17.5, and PD0.5 (**F**). Embryos were obtained from the same litters and analyzed at E15.5–PD0.5, respectively. Data are expressed as mean ± SD. Two-tailed Student’s *t*-test; *P*> 0.05 (ns), *P*< 0.01 (**), *P*< 0.001 (***). (**G**, **H**) Immunofluorescence staining of TUNEL in digested ovarian cells of Control and *Ehmt1^cKO^* embryos, MVH co-staining indicates the position of germ cells. DNA is stained with DAPI. Scale bar = 10 μm (**G**), and the number quantification for TUNEL-positive cells from Control and *Ehmt1^cKO^* embryos (**H**). Embryos were obtained from the same pregnant female and analyzed at E16.5. The data of quantitative analysis were obtained from four replicates of four embryos. Data are expressed as mean ± SD. Two-tailed Student’s *t*-test; *P* < 0.001 (***).

To investigate the reason for the failed ovulation in *Ehmt1^cKO^* mice, we observed the ovaries of adolescent mice. There was no significant difference in body weight between the two mice of group (Supplementary Fig. S2D), while *Ehmt1^cKO^* female mice had sharply smaller ovaries compared with the Control PD20 mice (Fig. 3A). So, the ovary-to-body weight ratio of *Ehmt1^cKO^* was remarkably lower than that of Control (Fig. 3B). We further performed immunofluorescence staining of the germ cell marker MVH to characterize the oocytes. In Supplementary Fig. S2E, we observed that the primordial follicles were almost depleted in PD20 *Ehmt1^cKO^* mice. The quantified numbers of follicles also confirmed that the depletion of primordial follicles was the main cause of infertility in *Ehmt1^cKO^* mice (Supplementary Fig. S2F).

When was the primordial follicles depleted? Before the primordial follicle pool was established, or later? Given that the primordial follicle pool is established within 3–5 days after birth [34], it was necessary to observe primordial follicles in PD5 mice. It was clear that PD5 *Ehmt1^cKO^* mice had the similar phenotype to that of PD20 *Ehmt1^cKO^* mice, characterized by severe depletion of primordial follicles (Fig. 3C and D). These data suggested that the scarcity of primordial follicles may be due to earlier germ cells loss.

To investigate the initial timepoint of germ cell loss in *Ehmt1^cKO^* mice, we performed immunofluorescence staining and cell counting at four developmental stages in embryonic ovaries. Figure 3E showed ovarian sections stained with anti-MVH antibodies at different developmental stages, and Fig. 3F summarized the ratio of germ cell numbers in Control VS *Ehmt1^cKO^* ovaries. Combining with the results, we concluded that there were only a quarter of germ cells (24.93%) preserved in *Ehmt1^cKO^* mice at the day of birth (PD0.5). The loss of germ cells (about 30%) began at E16.5 in *Ehmt1^cKO^* mice, comparing with Control. The percentage of germ cells was sharply decreased in *Ehmt1^cKO^* mice at E17.5 (above 65%). We performed TUNEL staining on ovarian sections from E15.5 and PD0.5 stages. Compared to the control group, a small accumulation of TUNEL signals was observed in EHMT1-deficient ovaries at E15.5, whereas a substantial accumulation of TUNEL signals was detected in the EHMT1-deficient ovaries at PD0.5 (Supplementary Fig. S2G). Subsequent high-resolution immunofluorescence staining revealed that these TUNEL signals were primarily localized to germ cells (Fig. 3G and H). In conclusion, these findings suggested that germ cell apoptosis was the primary cause of female infertility.

### EHMT1 deletion causes the arrest of zygotene stage due to unsuccessfully DSBs repair in female germ cells

**Figure 4.**
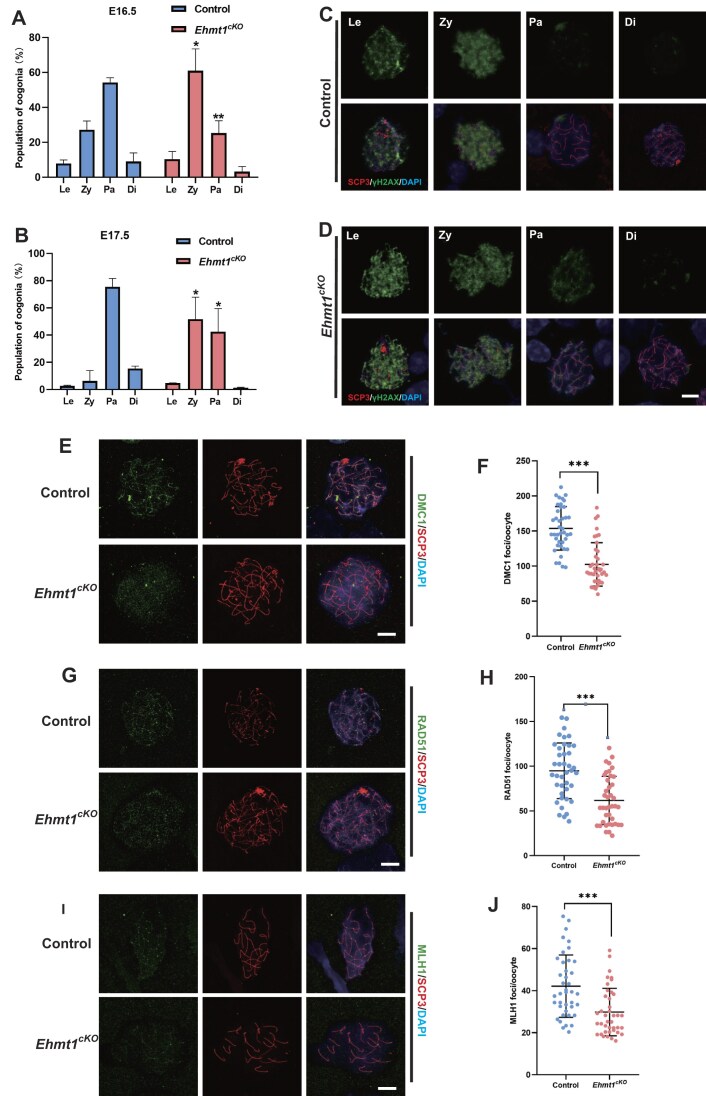
EHMT1-deleted female germ cells are defective in DNA DSBs repair. (**A**) Percentages of oogonia at the leptotene, zygotene, pachytene, and diplotene stages from Control (n = 3) and *Ehmt1^cKO^* (n = 3) embryos at E16.5. Data are expressed as mean ± SD. Two-tailed Student’s *t*-test; Leptotene (Le): *P* = 0.422176 (ns), Zygotene (Zy): *P* = 0.012219 (*), Pachytene (Pa): *P* = 0.002644 (**), Diplotene (Di): *P* = 0.151787 (ns). (**B**) Percentages of oogonia at the leptotene, zygotene, pachytene, and diplotene stages from Control (n = 3) and *Ehmt1^cKO^* (n = 3) embryos at E17.5. Data are expressed as mean ± SD. Two-tailed Student’s *t*-test; Leptotene (Le): *P* = 0.489851 (ns), Zygotene (Zy): *P* = 0.018414 (*), Pachytene (Pa): *P* = 0.033947 (*), Diplotene (Di): *P* = 0.470818 (ns). (**C**) Immunofluorescence staining of SYCP3 and γH2AX in chromosome spreads of Control female germ cells at various meiotic stages. Scale bar = 10 μm. (**D**) Immunofluorescence staining of SYCP3 and γH2AX in chromosome spreads of *Ehmt1^cKO^* female germ cells at various meiotic stages. Scale bar = 10 μm. (**E**, **F**) Immunofluorescence staining of SYCP3 and DMC1 in chromosome spreads of female germ cells from Control and *Ehmt1^cKO^* embryos. DNA labeled by DAPI. Scale bar = 10 μm (**E**). Quantification of DMC1 foci per cell in germ cells from Control (n = 40) and *Ehmt1^cKO^* (n = 40) embryos (**F**). Data are expressed as mean ± SD. Two-tailed Student’s *t*-test; *P* < 0.0001 (***). (**G**, **H**) Immunofluorescence staining of SYCP3 and RAD51 in chromosome spreads of germ cells from Control and *Ehmt1^cKO^* embryos. DNA labeled by DAPI. Scale bar = 10 μm (**G**). Quantification of RAD51 foci per cell in germ cells from Control (n = 40) and *Ehmt1^cKO^* (n = 40) embryos (**H**). Data are expressed as mean ± SD. Two-tailed Student’s *t*-test; *P* < 0.0001 (***). (**I**, **J**) Immunofluorescence staining of SYCP3 and MLH1 in chromosome spreads of germ cells from Control and *Ehmt1^cKO^* embryos. DNA labeled by DAPI. Scale bar = 10 μm (**I**). Quantification of MLH1 foci per cell in germ cells from Control (n = 40) and *Ehmt1^cKO^* embryos (**J**). Data are expressed as mean ± SD. Two-tailed Student’s *t*-test; *P* < 0.0001 (***).

Since the time of apoptosis was in meiotic prophase I, it was necessary to determine the developmental stage when meiosis was perturbed in *Ehmt1^cKO^* female germ cells. The previous studies have reported that the majority of female germ cells are in zygotene and pachytene stage at E16.5, and the majority of female germ cells are in pachytene stage at E17.5 [35, 36], so we suspected that the failure of the transition from zygotene to pachytene may cause germ cells to undergo apoptosis. The localization of EHMT1 was analyzed by female germ cells surface spreading. An antibody against SCP3, which is one of the SC components, was used to distinguish the stages of the female germ cells. The localization of EHMT1 was localized near the chromosome from leptotene to diplotene during female meiotic prophase I (Supplementary Fig. S3A). To further determine whether female germ cells could enter and successfully pass through meiotic prophase I, we summarized the frequency distribution of female germ cells at different developmental stages of embryonic ovaries. Further the quantification for the female germ cells of different developmental stages showed that the proportion of the zygotene cells was increased and that of pachytene cells was decreased in the *Ehmt1^cKO^* mice at E16.5 and E17.5 respectively (Fig. 4A and B). And surprisingly, the proportion of germ cells at each stage did not change significantly in *Ehmt1^cKO^* mice compared to Control groups at both E15.5 and D0.5 (Supplementary Fig. S3B and C).

Programmed DSBs repair are a crucial event during meiosis that facilitates chromosome synapsis and recombination [37, 38]. We first measured the efficiency of DSBs formation and repair by staining of γH2AX, Fig. 4C showed the signal of γH2AX in Control female germ cells of each stage, and there were obvious signals before zygotene due to unrepaired DSBs, and the signals gradually disappeared with the repair of DSBs in and after pachytene stage. Though there was no significant difference in γH2AX signals between Control and *Ehmt1^cKO^* female germ cells at diplotene, and both were maintained at very low levels, we found that a few *Ehmt1^cKO^* female germ cells still had obvious signal of γH2AX at pachytene stage (Fig. 4D). Multiple ssDNA-binding proteins protect ssDNA from recombinases by binding to the resected ssDNA [39]. To further assess the meiotic recombination process, we examined two DNA recombinases, DMC1 and RAD51, which drive strand invasion of homologous DNA double strands during prophase I of meiosis [13]. Additionally, MLH1 serves as a marker for crossovers during meiotic recombination [40]. We observed that DMC1 foci appeared along chromosome axes in Control female germ cell, but it was hardly found in *Ehmt1^cKO^* cells (Fig. 4E). Further quantification of the DMC1 foci in *Ehmt1^cKO^* female germ cell showed a distinctly low level compared with those of Control groups (Fig. 4F). Similarly, we observed that the distribution of RAD51 were reduced and the quantification of RAD51 foci were maintain a low level at the *Ehmt1^cKO^* groups (Fig. 4G and H). Furthermore, a notable decrease in MLH1 levels was observed in *Ehmt1^cKO^* female germ cells, suggesting that there were also defects in the crossover recombination process (Fig. 4I and J). These results demonstrated that EHMT1 plays a critical role in the programmed DSBs repair.

### EHMT1 deletion causes transcriptional dysregulation in female germ cells

**Figure 5.**
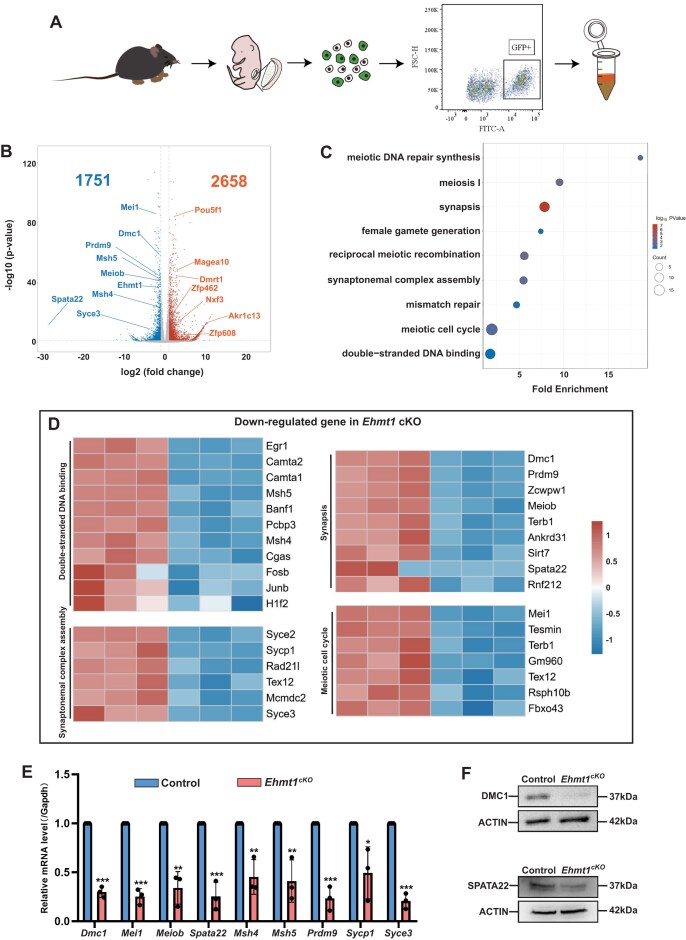
Downregulation of meiotic genes in EHMT1-deficient female germ cells. (**A**) Experimental workflow for collecting *Oct4*-eGFP positive cells: Isolation of E15.5 female embryos, gonadal dissection, preparation of single-cell suspensions, and FACS sorting of *Oct4*-eGFP positive cells. (**B**) Volcano plot of RNA-seq data from sorted *Oct4*-eGFP positive cells from ovaries of Control and *Ehmt1^cKO^* embryos, showing the number of DEGs (*P* < 0.05, fold change > 2). Significantly upregulated and downregulated genes in *Ehmt1^cKO^* female germ cells are highlighted respectively. DESeq2 Wald test *P*-values are calculated. (**C**) GO analysis of downregulated genes in *Oct4*-eGFP positive cells from *Ehmt1^cKO^* embryos. (**D**) Heatmap of gene expression for terms associated with downregulated genes identified by GO analysis in *Oct4*-eGFP-positive cells from *Ehmt1^cKO^* embryos. (**E**) RT-PCR analysis of downregulated meiotic genes in Control and *Ehmt1^cKO^* female germ cells. Data are expressed as mean ± SD. Two-tailed Student’s *t*-test; *P* < 0.05 (*), *P*< 0.01 (**), *P* < 0.001 (***). (**F**) Western blot analysis of DMC1 and SPATA22 in ovaries (E15.5) from Control and *Ehmt1^cKO^* embryos. Actin levels were used as loading controls. Each experiment was performed at least three times.

From E10.5 (PGC migrate to the genital ridge) to E13.5, oogonia are primarily in mitosis [5]. Meiosis begins at E13.5, with most oocytes entering the meiotic stage by E15.5 [6]. This process is accompanied with dramatic changes in gene expression. The loss of female germ cells and the arrest of meiosis both occurred at E16.5, rather than at E15.5, in the *Ehmt1^cKO^* embryos; therefore, we collected E15.5 female germ cells for transcriptomic analysis. We isolated germ cells from E15.5 embryos of female Tg (*Oct4*-eGFP) mice using FACS and performed RNA-seq (Fig. 5A). The method of isolating female germ cells had been validated in our previous work [41, 42].

RNA-seq analysis of the collected germ cells showed significant transcriptional dysregulation in EHMT1 deficient germ cells (Supplementary Fig. S4A and B). A total of 4409 DEGs were identified, with 2658 upregulated genes and 1751 downregulated genes (Fig. 5B). Overall, the upregulated genes in *Ehmt1^cKO^* female germ cells are primarily involved in “transcriptional regulation” and “cell death or apoptosis”, as confirmed by GO analysis (Supplementary Fig. S4C). Among the GO analysis of downregulated genes, several meiosis-related clusters were identified, including those involved in DSBs repair, meiotic progression, and synaptonemal complex assembly (Fig. 5C). Notably, several essential meiotic genes, including *Msh4*, *Msh5*, *Sycp1*, *Syce2*, *Syce3*, *Tex12*, *Dmc1*, *Meiob*, and *Spata22*, were significantly downregulated (Fig. 5D). RT-PCR validation of these meiotic genes confirmed significant downregulation of their mRNA levels in EHMT1 deficient female germ cells (Fig. 5E). Additionally, immunoblotting analysis showed that the protein expression of DMC1 and SPATA22 was significantly reduced in the ovaries of E15.5 *Ehmt1^cKO^* embryos (Fig. 5F). These results suggested that EHMT1 deletion causes dysregulation of transcription and downregulation of essential meiotic genes, which might account for the arrest of meiosis and loss of female germ cells.

### EHMT1 deletion disrupts the re-establishment of H3K9me2 in female germ cells and upregulates the transcriptional repressors

**Figure 6.**
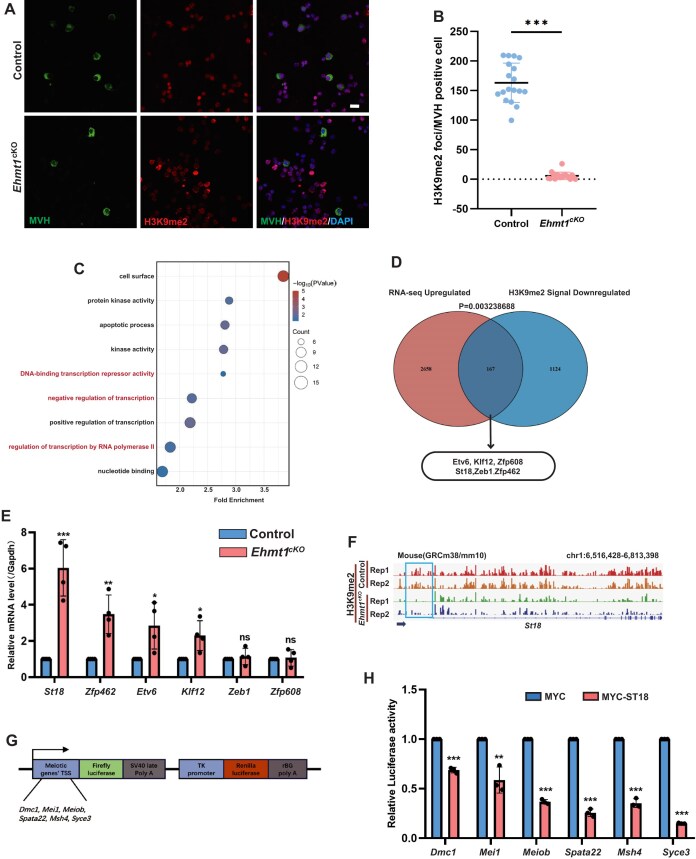
Absence of H3K9me2 leads to aberrant expression of transcriptional regulators, thereby disrupting the transcription of meiotic gene. (**A** and **B**) Immunofluorescence staining of H3K9me2, MVH , and DAPI in ovarian cells from Control (n = 4) and *Ehmt1^cKO^* (n = 4) embryos (E15.5) (**A**), and fluorescence quantification for H3K9me2 in MVH-positive cells from Control and *Ehmt1^cKO^* embryos (**B**). Embryos were obtained from the same pregnant female. The data of quantitative analysis were obtained from 20 replicates of 3 embryos. Scale bar = 10 μm. Data are expressed as mean ± SD. Two-tailed Student’s *t*-test; *P* < 0.0001 (***). (**C**) GO analysis integrating upregulated genes in *Oct4*-eGFP-positive cells (RNA-seq) and H3K9me2 signal downregulated genes in SSEA1-positive cells (H3K9me2 Cut&Tag-seq) from *Ehmt1^cKO^* embryos. (**D**) Venn diagram showing the overlap of upregulated genes in *Oct4*-eGFP positive cells (RNA-seq) and H3K9me2 signal downregulated genes in SSEA1-positive cells (H3K9me2 Cut&Tag-seq) from *Ehmt1^cKO^* embryos. Six transcription factors from the transcriptional repression pathway are annotated. Choosing all the coding genes in mice as the background set for statistical testing by using the hypergeometric distribution, and *P*= .003238688 (**). (**E**) RT-PCR analysis of six transcription factors in Control and *Ehmt1^cKO^* female germ cells. Data are expressed as mean ± SD. Two-tailed Student’s *t*-test; *P* > 0.05 (ns), *P* < 0.05 (*), *P* < 0.001 (**). *P* < 0.0001 (***). (**F**) Genome browser tracks depicting reads accumulation of H3K9me2 on transcription factors *St18* in Control and *Ehmt1^cKO^* SSEA1-positive cells using IGV software. (**G**) Schematic diagram of the dual-luciferase reporter construct: The TSS of the meiotic gene drives the transcription of *Firefly luciferase*, while the *TK promoter* drives the transcription of *Renilla luciferase*. (**H**) Dual-luciferase reporter gene assays showed that when ST18 was overexpressed, the luciferase activity of the meiotic gene promoter regions in HEK293T cells was significantly decreased respectively. Data are expressed as mean ± SD. Two-tailed Student’s *t*-test; *P* < 0.01 (**). *P* < 0.001 (***).

As a H3K9 methyltransferase, EHMT1 mediates the re-establishment of H3K9me2 during meiosis (Fig. 1A and B). To investigate the mechanism underlying the downregulation of meiotic genes, we focused on the histone modifications and examined the distribution of H3K9me2 in EHMT1 deficient female germ cells. Immunofluorescence staining of ovarian cells revealed that, compared to the Control group, H3K9me2-positive germ cells were nearly absent in EHMT1-deficient mice (Fig. 6A and B). In contrast, both the number of H3K27me3-positive germ cells and fluorescence intensity of H3K27me3 showed no significant differences between Control and EHMT1-deficient mice (Supplementary Fig. S5A and B), a similar pattern was also observed for H3K9me1 (Supplementary Fig. S5C and D). H3K9me2 plays a crucial role in gene expression, especially in inhibiting transcription of genes [43, 44], we also found that more genes were upregulated than downregulated in *Ehmt1^cKO^* female germ cells (Fig. 5B). Therefore, it is necessary to explore how the absence of H3K9me2 impact the genomic landscape of histone modifications in female germ cells. To achieve this, we employed the method shown in Fig. 1E to sort the germ cells and performed Cut&Tag sequencing.

With the high reproducibility of the Cut&Tag analysis (Supplementary Fig. S5E), we found a reasonable decrease in the level of H3K9me2 at differentially modified regions (Supplementary Fig. S5F and G), which was consistent with the absence of H3K9me2 in *Ehmt1^cKO^* germ cells (Fig. 6A and B). Since H3K9me2 inhibits gene transcription, we first examined whether the downregulation of the meiotic genes was due to abnormal enrichment of H3K9me2 near the gene. Surprisingly, H3K9me2 was normally enriched at the meiotic genes (*Dmc1*, *Spata22*, *Syce3*) and was maintained at very low levels (Supplementary Fig. S6A). To further understand the causes of downregulation of the meiotic genes, we performed an overlap analysis between the “H3K9me2 signal downregulated” genes and the “RNA-seq upregulated” genes, identifying a total of 167 enriched genes (Fig. 6D). In addition, we performed statistical testing using the hypergeometric distribution, choosing all the coding genes in mice as the background set. The *P*-value calculated was 0.003238688. GO analysis revealed that the overlapping genes were primarily enriched in processes such as apoptosis, transcription regulation, and transcriptional repression by DNA-binding factors (Fig. 6C). Interestingly, we identified several transcriptional repressors, including *Etv6* and zinc-finger protein family members such as *Klf12*, *Zfp608*, *Zfp462*, *St18*, and *Zeb1*. We hypothesize that H3K9me2 modification may regulate the expression of these transcription factors, which might function in meiotic gene transcription. To further confirm the expression levels of these transcription factors, we performed RT-PCR. The results showed that most of the transcription factors (*St18*, *Etv6*, *Zfp462*, and *Klf12*) were significantly upregulated in EHMT1-deficient female germ cells (Fig. 6E). Meanwhile, the enrichment of H3K9me2 on ST18 (Fig. 6F) and other transcription factors (Supplementary Fig. S6B) was lost to varying degrees in EHMT1-deficient female germ cells. These results indicated that the absence of H3K9me2 might lead to increased expression of transcriptional repressors such as *St18*, *Etv6*, *Zfp462*, and *Klf12*.

### ST18 and ZFP462 regulate the transcription of meiotic gene

Given that the transcriptional repressors negatively regulate gene transcription [45, 46], we hypothesized that upregulated transcriptional repressors may affect the transcription of meiotic genes. We then conducted luciferase reporter assays. We first constructed the overexpression vectors of *Myc-Etv6*, *Myc-St18*, and *Myc-Zfp462* respectively, as well as luciferase reporter constructs for *Dmc1*, *Mei1*, *Meiob*, *Spata22*, *Msh4*, and *Syce3* respectively (Fig. 6G). After successful expression in HEK293T cells (Supplementary Fig. S6C), we observed that overexpression of ST18 significantly reduced luciferase activity for all six meiosis-related genes (Fig. 6H), whereas overexpression of ZFP462 caused a mild decrease in luciferase activity for *Dmc1* and *Spata22* (Supplementary Fig. S6D). Overexpression of ETV6 did not affect any of the reporter activities (Supplementary Fig. S6E). These results suggested that ST18 negatively regulates the transcription of meiotic genes, while ZFP462 has a limited role in regulating the transcription of specific meiotic genes like *Dmc1* and *Spata22*.

**Figure 7.**
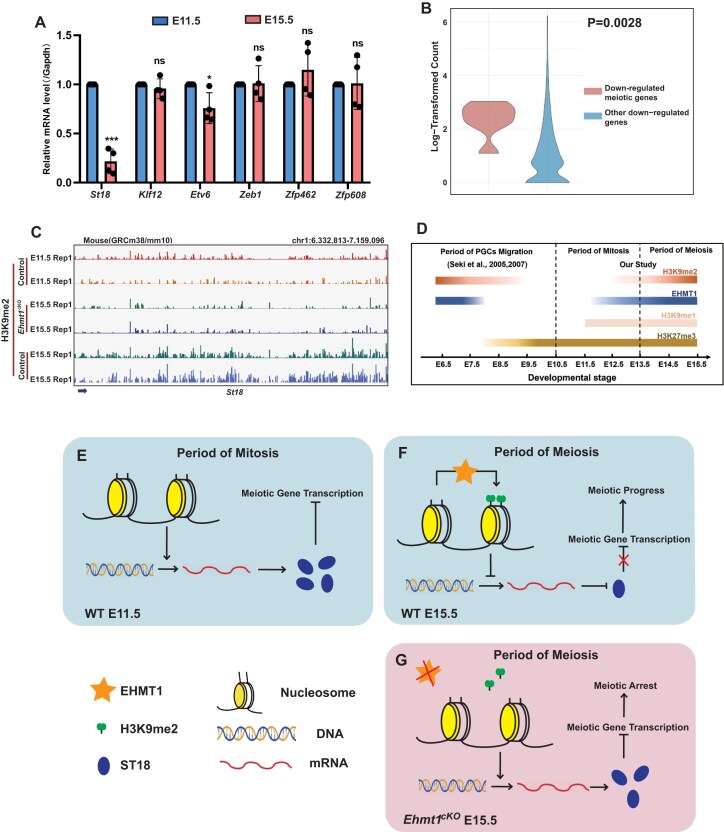
Repression of ST18 expression by H3K9me2 is a prerequisite for the transcription of a subset of meiotic genes. (**A**) RT-PCR analysis of the upregulated transcription factors in mitosis (E11.5) and meiosis (E15.5) female germ cells. Data are expressed as mean ± SD. Two-tailed Student’s *t*-test; *P* > 0.05 (ns), *P* < 0.001 (***). (**B**) The Log-Transformed Counts of the ST18 binding motif “AAAGTTT” in Downregulated meiotic genes versus Other downregulated genes. Statistical differences between the groups were assessed using the Wilcoxon rank-sum test (one-tailed), *P*= 0.0028 (**). (**C**) Genome browser tracks depicting reads accumulation of H3K9me2 on transcription factors *St18* in Control (E11.5) and *Ehmt1^cKO^* (E11.5 and E 15.5) SSEA1-positive cells using IGV software. (**D**) Cellular dynamics of H3K9me2, H3K9me1, H3K27me3, and EHMT1 in PGCs before [3, 4] and after colonization in embryos. (**E**) Schematic working model of the H3K9me2 absence-mediated expression of ST18 restricts the transcription of the meiotic genes in WT(wild type) oogonia at E11.5. (**F**) Schematic working model of the H3K9me2-mediated downregulation of ST18 elease restrict the transcription of the meiotic genes in oogonia at E15.5. (**G**) Schematic working model of the H3K9me2 absence-mediated upregulation of ST18 inhibit the transcription of the meiotic genes in *Ehmt1^cKO^* oogonia at E15.5.

H3K9me2 is absent in mitosis (E11.5) but re-established in meiosis (E15.5) (Fig. 1A). In addition, we demonstrated the strong restriction of ST18 on transcription of meiotic genes through H3K9me2 deletion model. We speculated that the absence of H3K9me2 in mitosis (E11.5) allows for the expression of transcriptional suppressors, thus limiting the transcription of meiotic genes and controlling the timing of meiosis progression. To verify this, we collected female germ cells from E11.5 and E15.5 embryos respectively for RT-PCR analysis, and the results showed that the mRNA level of *St18* and *Etv6* in E11.5 was significantly higher than that in E15.5, while the mRNA levels of *Klf12*, *Zfp462*, *Zeb1*, and *Zfp608* were not significantly different (Fig. 7A). H3K9me2 may negatively regulates *St18* to orchestrate meiotic progression of female germ cells by controlling transcription of key meiotic genes.

A previous report found that ST18 specifically binds to the “AAAGTTT” motif, which is located upstream of the thymidine kinase promoter, and suppresses transcription activity of the reporter construct [47]. To demonstrate the specificity of ST18 in the regulation of meiotic genes, we examined the distribution of the ST18 binding motif “AAAGTTT” within the upstream regulatory regions of each of the six downregulated meiotic genes and their upstream gene bodies and the 1.0 kb upstream regulatory regions of six downregulated meiotic genes (Supplementary Fig. S6F). Next, we performed a statistical comparison of the “AAAGTTT” motif counts of six downregulated meiotic genes with other downregulated genes using the Wilcoxon rank-sun test, and found that the distribution of the “AAAGTTT” motif counts of six downregulated meiotic genes was significantly higher than that of other genes in the genome (Fig. 7B). In addition, we compared the reads accumulation of H3K9me2 on the transcription factor ST18 in female germ cells from wild-type E11.5 mice, Control E15.5 mice, and EHMT1-deficient E15.5 mice, we found that the distribution pattern of H3K9me2 on ST18 in wild-type E11.5 mice was highly similar to that observed in EHMT1-deficient E15.5 female germ cells, both showed a marked reduction in H3K9me2 abundance compared to the Control E15.5 female germ cells. (Fig. 7C). This suggests that the upregulation of ST18 at E11.5 is associated with the low levels of H3K9me2 accumulation, a pattern that resembles that of EHMT1-deficient E15.5 female germ cells. The above results indicate that ST18 is specific for the transcriptional regulation of meiotic genes.

## Discussion

Epigenetic reprogramming is widespread and highly active during gametogenesis and maturation, involving processes such as DNA methylation and histone modifications [48]. The dynamic changes in DNA methylation during gametogenesis are relatively well characterized, with distinct reprogramming patterns observed in male and female germ cells after sexual differentiation [2]. Histone modifications, another important type of epigenetic regulation, have been less studied in the context of gametogenesis. Previous studies by Saitou group described dynamic changes in histone modifications in PGCs prior to their colonization and emphasized the potential importance of the elimination of H3K9me2 for the developmental maturation of PGCs [3, 4]. However, sexual differentiation begins immediately after PGC colonization in the gonads, with female germ cells proceeding into meiosis shortly afterward. This raises the question that how H3K9me2 regulate germ cells to enter meiosis. Previous studies have shown that H3K9me2 is present in germ cells during meiosis, specifically, in male germ cells before the pachytene stage and throughout the prophase of meiosis in female germ cells [20]. These findings suggest that H3K9me2 is re-established after PGC colonization, and it likely plays different roles during meiosis in males and females. In this study, we first examined the expression of H3K9me2 in female germ cells at three key time points: Mitosis stage after colonization (E11.5), Mitosis-Meiosis transition stage (E13.5), Meiosis stage (E15.5). We found that H3K9me2, rather than H3K27me3 or H3K9me1, was gradually re-established during this process. Interestingly, the re-establishment of H3K9me2 occurred later than the EHMT1 expression (Fig. 7D). These observations suggested that EHMT1 may play critical roles in the re-establishment of H3K9me2 prior to the onset of meiosis, and that this modification is involved in key biological processes during meiosis.

Histone modifications during meiosis have been extensively studied. Prdm9, an H3K4 methyltransferase, was the first gene linked to initiating recombination hotspots in mammals, and Prdm9 KO severely impairs female fertility [19]. Another H3K9 methyltransferase, G9a (also known as EHMT2), plays a crucial role in HR during meiosis. G9a depletion in mice leads to massive oocyte loss and significantly reduces the fertility [20]. In our study, we demonstrate that EHMT1 is involved in the re-establishment of H3K9me2 and explore its critical function during the early stages of female meiosis. Using a *Tnap*-Cre mediated conditional KO mouse model (in which Cre recombinase is expressed in PGCs as early as E9.5) [33], we conditionally deleted EHMT1 in female germ cells, which resulted in a severe restriction of progression in meiosis. Our findings emphasize that EHMT1-mediated H3K9me2 modification plays a key physiological role in maintaining gene expression balance during female germ cell meiosis. Furthermore, we observed the transcriptional regulation of critical meiotic genes depends on the expression of the transcription factor ST18, thus expanding our understanding of how histone modifications contribute to the progression of female meiosis.

Further transcriptomic analysis and Cut&Tag seq analysis revealed that many transcriptional repressive factors were upregulated in EHMT1 deficient female germ cells, and RT-PCR confirmed their increased expression. These factors have previously been reported to function as transcriptional repressors [28, 29]. This suggests the presence of a balance mechanism, in which the upregulation of transcriptional repressors may inhibit the transcription of meiotic genes, leading to meiotic arrest. Immunofluorescence staining also showed that H3K9me2 gradually accumulates as germ cells enter meiosis. We hypothesize that the absence of H3K9me2 during mitosis (E11.5) in germ cells leads to upregulation of ST18 expression, which suppresses the transcription of meiotic genes (Fig. 7E). During the initiation of meiosis, the establishment of H3K9me2 suppresses ST18 expression, allowing meiosis to proceed smoothly (Fig. 7F). In EHMT1 deficient female germ cells, the failure of H3K9me2 re-establishment results in upregulation of ST18, which is similar to that in mitosis (E11.5) (Fig. 7G). RT-PCR analysis of germ cells at mitosis (E11.5) and meiosis (E15.5) confirmed a dramatic downregulation of ST18 at E15.5. And the abundance analysis of H3K9me2 at E11.5 and E15.5 showed a significant accumulation of H3K9me2 on the ST18 gene at E15.5. These findings supported our hypothesis that H3K9Mme2-mediated ST18 expression regulates meiotic gene transcription, thus promoting meiosis progression.

ST18 and ZFP462 are both zinc finger proteins that bind directly to DNA and regulate gene transcription [28, 29]. ST18 is a tumor suppressor gene, and its family members have been extensively studied for their roles in transcriptional regulation [49]. In our study, luciferase reporter assays confirmed that ST18 suppresses the expression of several meiotic genes, including *Dmc1*, *Mei1*, *Meiob*, *Spata22*, *Msh4*, and *Syce3*. ST18 expression was significantly upregulated during both mitosis (E11.5) and in EHMT1 deficient meiotic female germ cells (E15.5), indicating its role in repressing the transcription of meiotic genes. Recent study suggested that during stem cell differentiation, ZFP462 recruits GLP(EHMT1)/G9A(EHMT2) to establish heterochromatin, thereby limiting DNA accessibility and transcription factor binding [29]. In female germ cells during meiosis, ZFP462 expression is suppressed by H3K9me2 to prevent disruptions in the transcription of meiotic genes. Compared to ST18, the repressive effect of ZFP462 on meiotic gene transcription is relatively lower, affecting only a few genes, such as *Dmc1* and *Spata22*. We hypothesize that there may be two reasons: (i) most meiotic gene transcription is not regulated by ZFP462, and (ii) our luciferase reporter system did not include the full enhancer regions of these genes, due to ZFP462 has been shown to primarily bind the enhancer regions in ESCs, with a smaller fraction binding to transcription start sites [29]. In this study, we focused on the role of ST18 in the transcriptional regulation of meiotic genes, rather than ZFP462. On one hand, ST18 exhibited more significant differences in expression levels and H3K9me2 enrichment at E11.5 and E15.5. On the other hand, ZFP462 did not exhibited significant differences in expression levels and did not demonstrate a more prominent role in regulating meiotic gene transcription.

Meiosis is a critical step in gametogenesis. Our study highlights the role of EHMT1-mediated H3K9me2 in regulating the expression of critical meiotic genes during female meiosis. Additionally, we emphasize the crucial role of transcriptional repressors, particularly ST18, in maintaining the transcriptional balance of meiotic genes when H3K9me2 is reprogramming. This study enhances our understanding of how histone modifications and transcription factors work together to stabilize meiotic gene transcription during female meiosis.

## Supplementary Material

gkaf657_Supplemental_File

## Data Availability

The authors declare that all data supporting the findings of this study are available within the article and its appendix files or from the corresponding author upon reasonable request. All raw sequencing data were deposited in the National Genomics Data Center (NGDC) Genome Sequence Archive (GSA) database: Raw data of H3K9me2 Cut&Tag analysis for E15.5 female germ cells (Accession number is CRA021737); Raw data of transcriptomics for E15.5 female germ cells (Accession number is CRA021738). All processed data were deposited in the National Genomics Data Center (NGDC) Open Archive for Miscellaneous Data (OMIX) database: Processed data of H3K9me2 Cut&Tag analysis for E15.5 female germ cells (Accession number is OMIX008517); Processed data of transcriptomics analysis for E15.5 female germ cells (accession number is OMIX008506).
