# Correction to ‘Re-establishment of H3K9me2 eliminates the transcriptional inhibition of ST18 on meiotic genes and orchestrates female germ cell development’

**DOI:** 10.1093/nar/gkaf979

**Published:** 2025-09-27

This is a correction to: Bing-Wang Zhao, Yi-Na Zhang, Tie-Gang Meng, Yuan-Hong Xu, Yi-Ke Lu, Si-Min Sun, Jia-Ni Guo, Xue-Mei Yang, Zhen-Bo Wang, Re-establishment of H3K9me2 eliminates the transcriptional inhibition of ST18 on meiotic genes and orchestrates female germ cell development, Nucleic Acids Research, Volume 53, Issue 13, 22 July 2025, gkaf657, https://doi.org/10.1093/nar/gkaf657

Supplementary Tables 1 and 2 were accidentally omitted at revision. The correct Supplementary data is now provided.

This change does not affect the results, discussion and conclusions presented in the article. The published article has been updated.

