# Supplementary material for: Re-establishment of H3K9me2 eliminates the transcriptional inhibition of ST18 on meiotic genes and orchestrates female germ cell development
Source: Nucleic Acids Res. 2025 Jul 12;53(13):gkaf657. doi: 10.1093/nar/gkaf657 (PMC12255295; doi:10.1093/nar/gkaf657)
Supplement: gkaf657_Supplemental_File [file gkaf657_supplemental_file.pdf]

Supplementary Figure S1

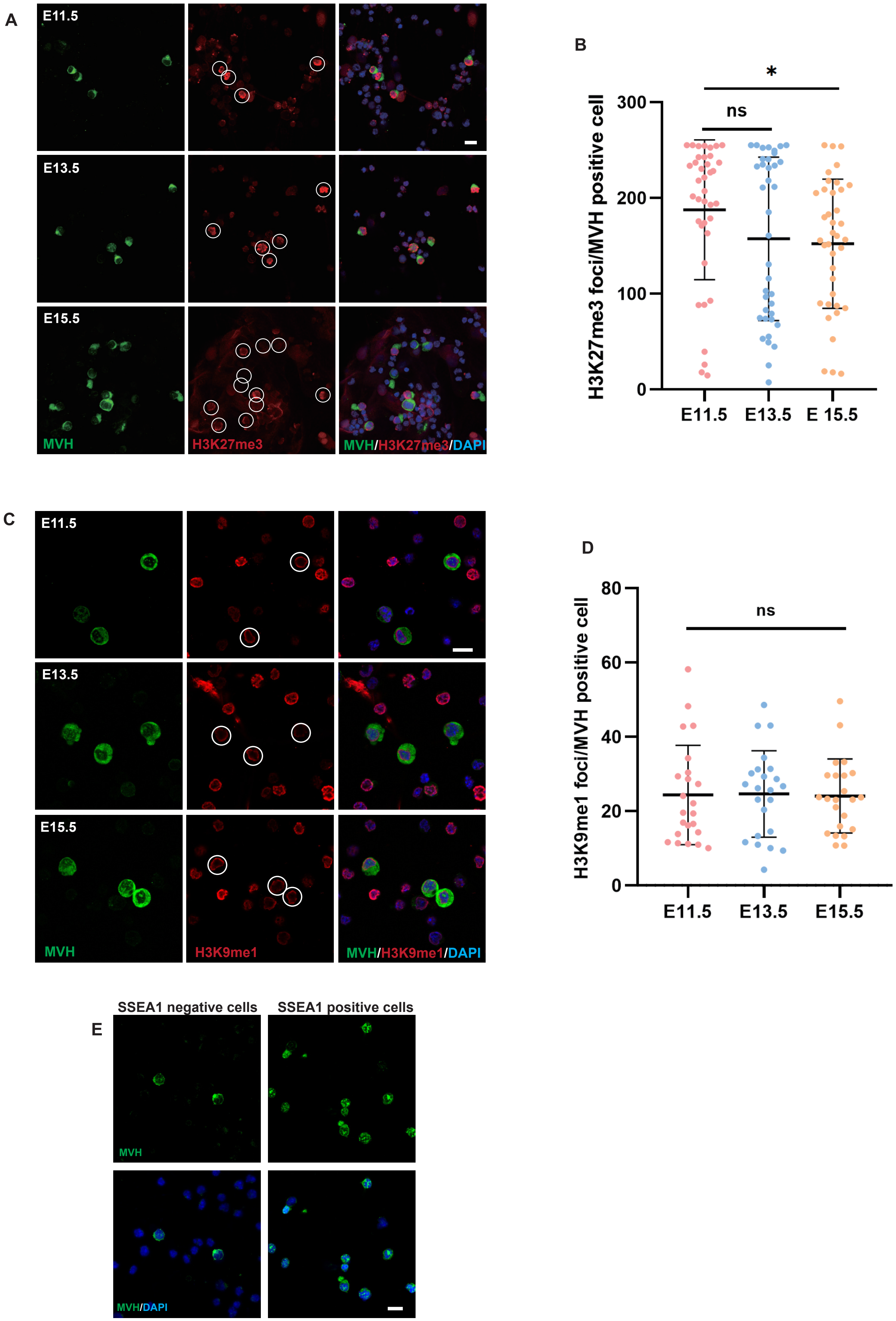

Supplementary Figure S1.

(**A** and **B)** Immunofluorescence staining of ovarian cells at E11.5, E13.5 and E15.5 using anti-H3K27me3 (red), anti-MVH (green) and DAPI (blue). Dashed circles represent germ cells (**A**), and fluorescence quantification for H3K27me3 in MVH-positive cells (**B**), the data of quantitative analysis were obtained from 20 replicates. Scale bar = 10 μm. Two-tailed Student's t-test; $P < 0.05$ (*). (**C** and **D)** Immunofluorescence staining of ovarian cells at E11.5, E13.5 and E15.5 using anti-H3K9me1 (red), anti-MVH (green) and DAPI (blue). Dashed circles represent germ cells (**C**), and fluorescence quantification for H3K9me1 in MVH-positive cells (**D**), the data of quantitative analysis were obtained from 20 replicates. Scale bar = 10 μm. Two-tailed Student's t-test. (**E**) Immunofluorescence staining of MVH (Green) on SSEA1 positive cells and SSEA1 negative cells. Nuclei were stained with DAPI. Scale bars = 10 μm.

Supplementary Figure S2

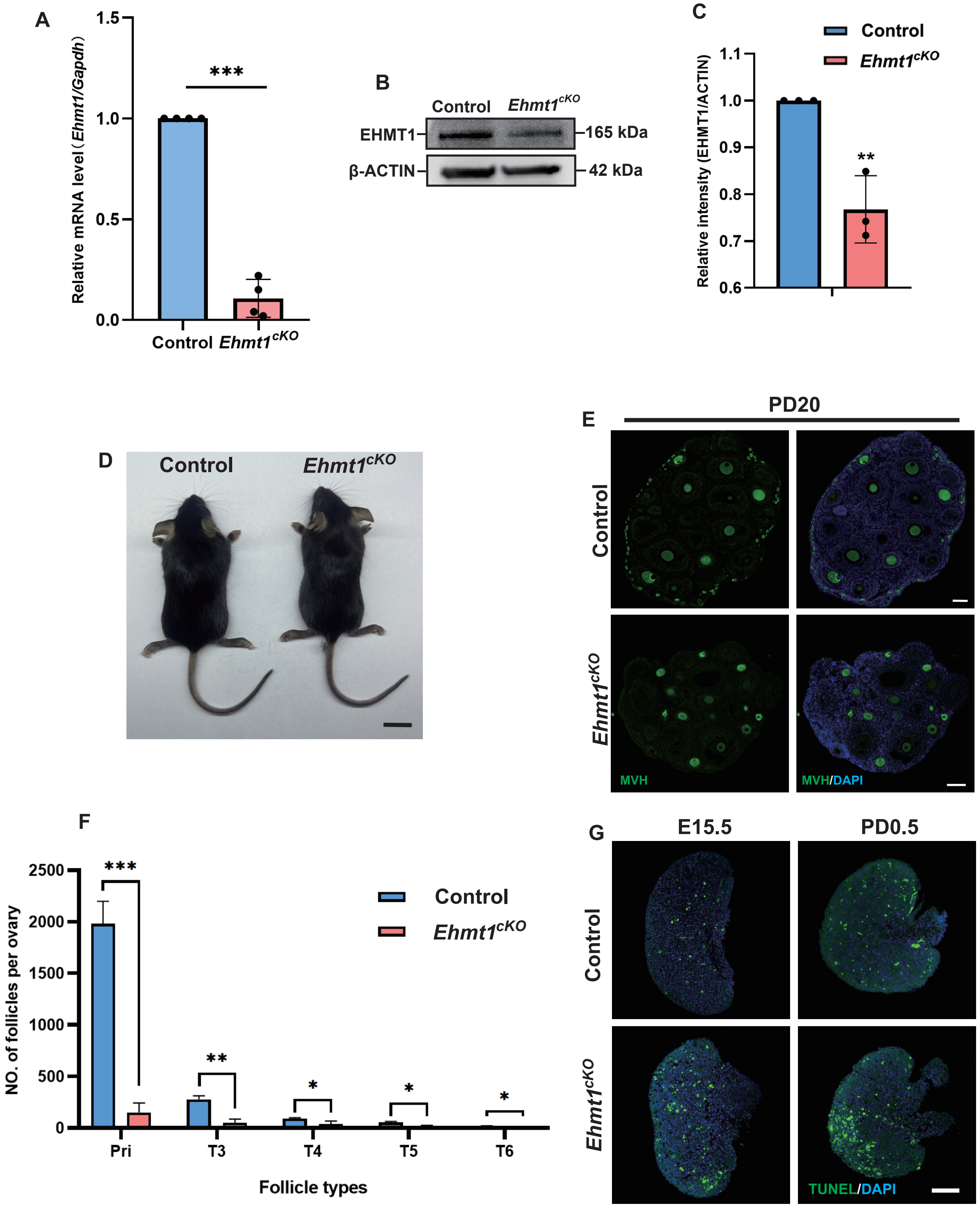

Supplementary Figure S2. EHMT1 depletion cause the loss of primordial follicles. （**A**）RT-PCR analysis of EHMT in Control and *Ehmt1*^{cKO} female germ cells. Data are expressed as mean ± SD. Two-tailed Student's t-test; $P < 0.01$ (**). (**B** and **C**) Western blot analysis of EHMT1 protein in ovaries from Control and *Ehmt1*^{cKO} embryos at E13.5, actin is used as a loading control (**B**), and the data of quantitative analysis were obtained from 3 times independent repeats (**C**). Data are expressed as mean ± SD. Two-tailed Student's t-test; $P < 0.01$ (**). (**D**) Comparison of morphological features between Control (n=4) and *Ehmt1*^{cKO} (n=4) females. Mice were born in the same litters and analyzed at PD20. Scale bar = 1 cm. (**E**) Immunofluorescence staining of MVH (green) in Control and *Ehmt1*^{cKO} ovaries, with DNA labeled by DAPI (blue). Mice were born in the same litter and analyzed at PD20. Scale bar = 100 μm. (**F**) Follicle counts in Control (n=3) and *Ehmt1*^{cKO} (n=3) ovaries at PD20. Primordial: Primordial Follicles, T3: Type 3 Follicles, T4: Type 4 Follicles, T5: Type 5 Follicles, T6: Type 3 Follicles; Data are expressed as mean ± SD. Two-tailed Student's t-test; $P < 0.05$ (*), $P < 0.01$ (**), $P < 0.001$ (***). (**G**) TUNEL (green) staining of ovaries from Control (n=3) and *Ehmt1*^{cKO} (n=3), with DNA labeled by DAPI (blue). Embryos were obtained from the same litters and analyzed at E15.5 and PD0.5 respectively. Scale bar = 25 μm.

Supplementary Figure S3

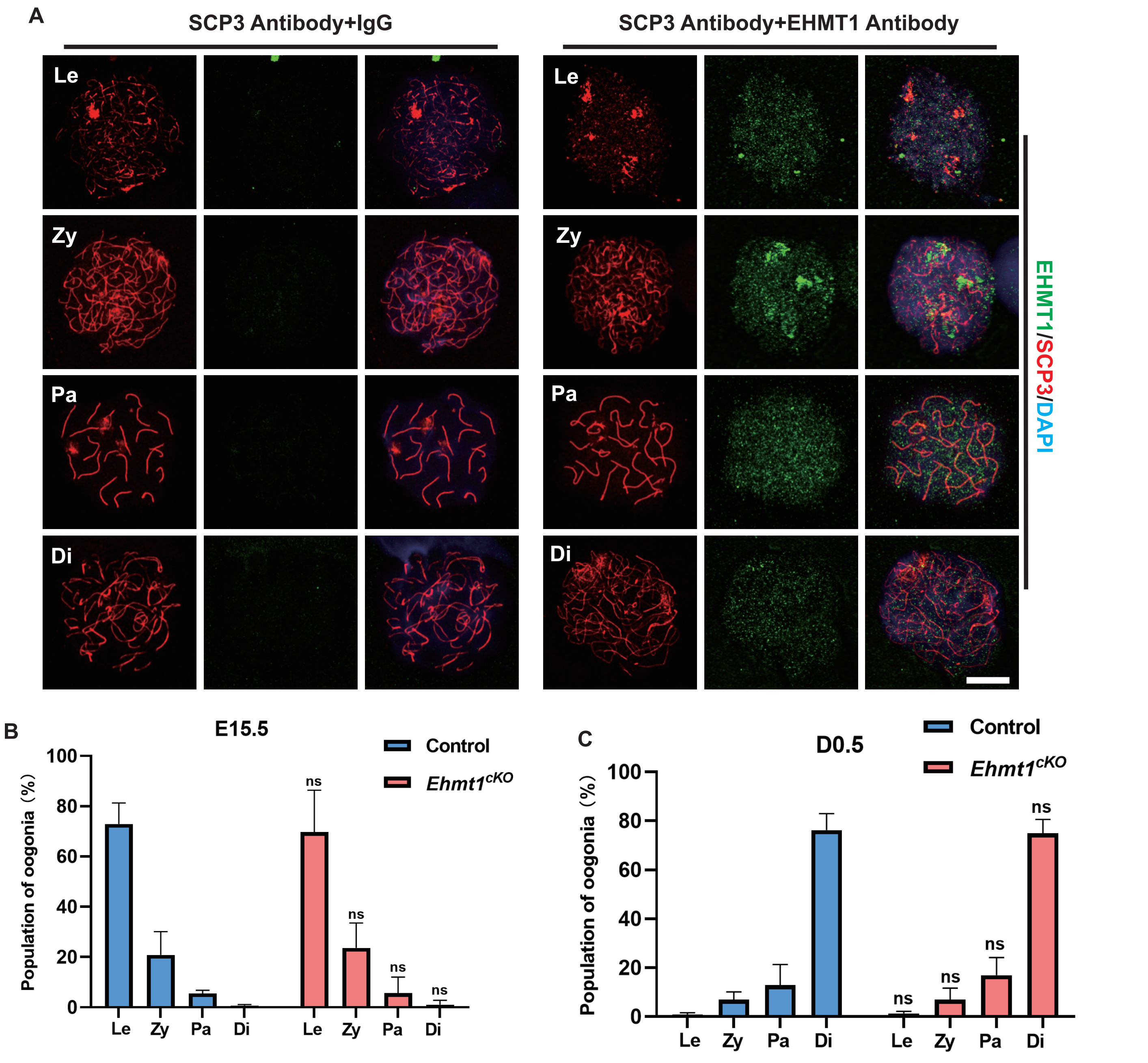

Supplementary Figure S3. Normal rates of female germ cells at different frequency of meiotic prophase I stages in *Ehmt1^{cKO}* at E15.5 and PD0.5 respectively. (**A**) Immunofluorescence staining of EHMT1 or IgG (green), SYCP3 (red) in chromosome spreads of female germ cells at various meiotic prophase I stages, with DNA labeled by DAPI (blue). Scale bar = 10 μm. Leptotene (Le), Zygotene (Zy), Pachytene (Pa), Diplotene (Di). (**B**) Percentages of oogonia at the leptotene, zygotene, pachytene, and diplotene stages from Control (n=3) and *Ehmt1^{cKO}* (n=3) embryos at E15.5. Data are expressed as mean ± SD. Two-tailed Student's t-test; Leptotene (Le): *P* = 0.776388 (ns), Zygotene (Zy): *P* = 0.745204 (ns), Pachytene (Pa): *P* = 0.952853 (ns), Diplotene (Di): *P* = 0.748810 (ns). (**C**) Percentages of oogonia at the leptotene, zygotene, pachytene, and diplotene stages from Control (n=3) and *Ehmt1^{cKO}* (n=3) embryos at PD0.5. Data are expressed as mean ± SD. Two-tailed Student's t-test; Leptotene (Le): *P* = 0.608982 (ns), Zygotene (Zy): *P* >0.999999 (ns), Pachytene (Pa): *P* = 0.562636 (ns), Diplotene (Di): *P* = 0.816782 (ns).

Supplementary Figure S4

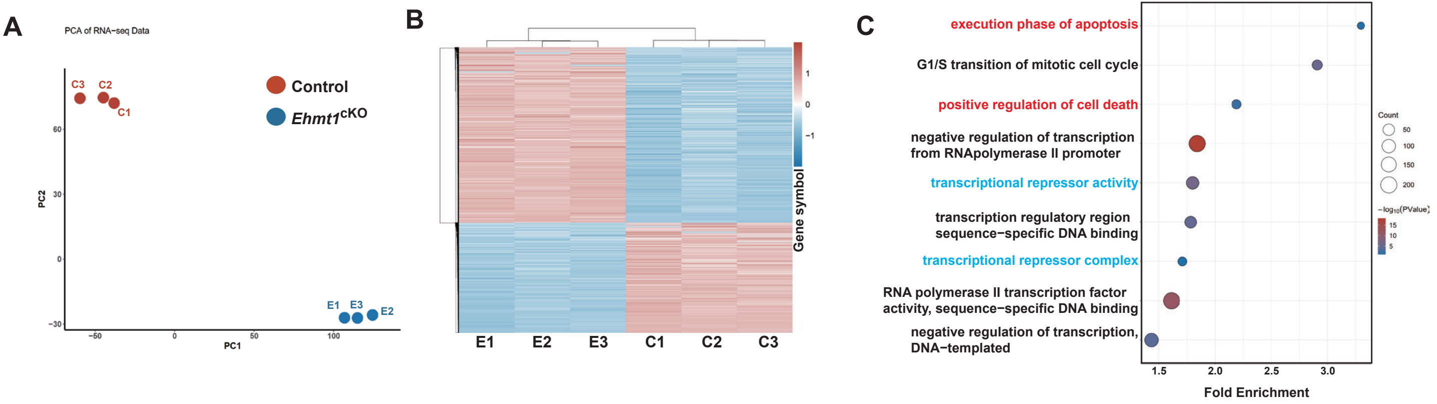

Supplementary Figure S4. Upregulated Genes in *Ehmt1^{cKO}* female germ cells are primarily involved in transcriptional regulation. (**A**) Principal component analysis (PCA) of RNA-seq data from Control and *Ehmt1^{cKO}* *Oct4*-eGFP positive cells. (**B**) Heatmap showing the expression levels of genes in Control and *Ehmt1^{cKO}* *Oct4*-eGFP positive cells. (**C**) Gene Ontology (GO) analysis of upregulated genes in *Oct4*-eGFP positive cells from *Ehmt1^{cKO}* embryos.

Supplementary Figure S5

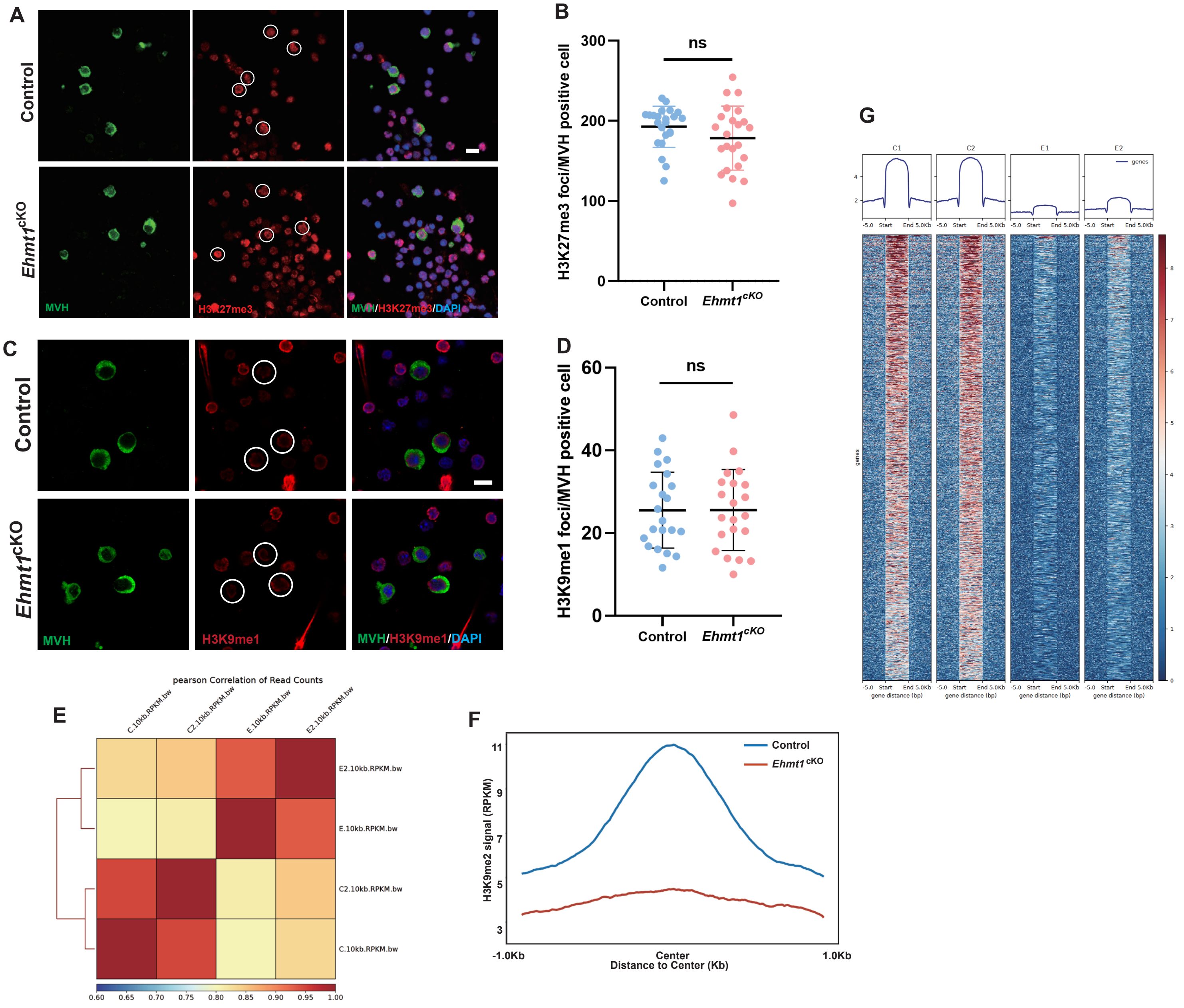

Supplementary Figure S5. H3K9me2 but not H3K27me3 was significantly decreased in EHMT1-deficient female germ cells (**A** and **B**) Immunofluorescence staining of H3K27me3 (red), MVH (green), and DAPI (blue) in ovarian cells from Control and *Ehmt1*^{cKO} embryos (E15.5), scale bar = 10 μm (**A**), and fluorescence quantification for H3K27me3 in MVH-positive cells (**B**). The data of quantitative analysis were obtained from 20 independent repeats Data are expressed as mean ± SD. Two-tailed Student's t-test; P >0.05 (ns). (**C** and **D**) Immunofluorescence staining of H3K9me1 (red), MVH (green), and DAPI (blue) in ovarian cells from Control and *Ehmt1*^{cKO} embryos (E15.5), scale bar = 10 μm (**C**), and fluorescence quantification for H3K9me1 in MVH-positive cells (**D**). The data of quantitative analysis were obtained from 20 independent repeats Data are expressed as mean ± SD. Two-tailed Student's t-test; P >0.05 (ns). (**E**) Correlation heatmap of CUT&Tag replicates for the H3K9me2 histone modification in Control (C1 and C2) and *Ehmt1*^{cKO} (E1 and E2) SSEA1 positive cells. (**F**) Average H3K9me2 Cut&Tag signals of SSEA1-positive cells from Control and *Ehmt1*^{cKO} embryos within -1 kb/+1 kb of gene body. (**G**) Heatmap of H3K9me2 signal levels in SSEA1-positive cells from Control and *Ehmt1*^{cKO} embryos on the genomic regions between −5.0 kb upstream and +5.0 kb downstream of gene body.

Supplementary Figure S6

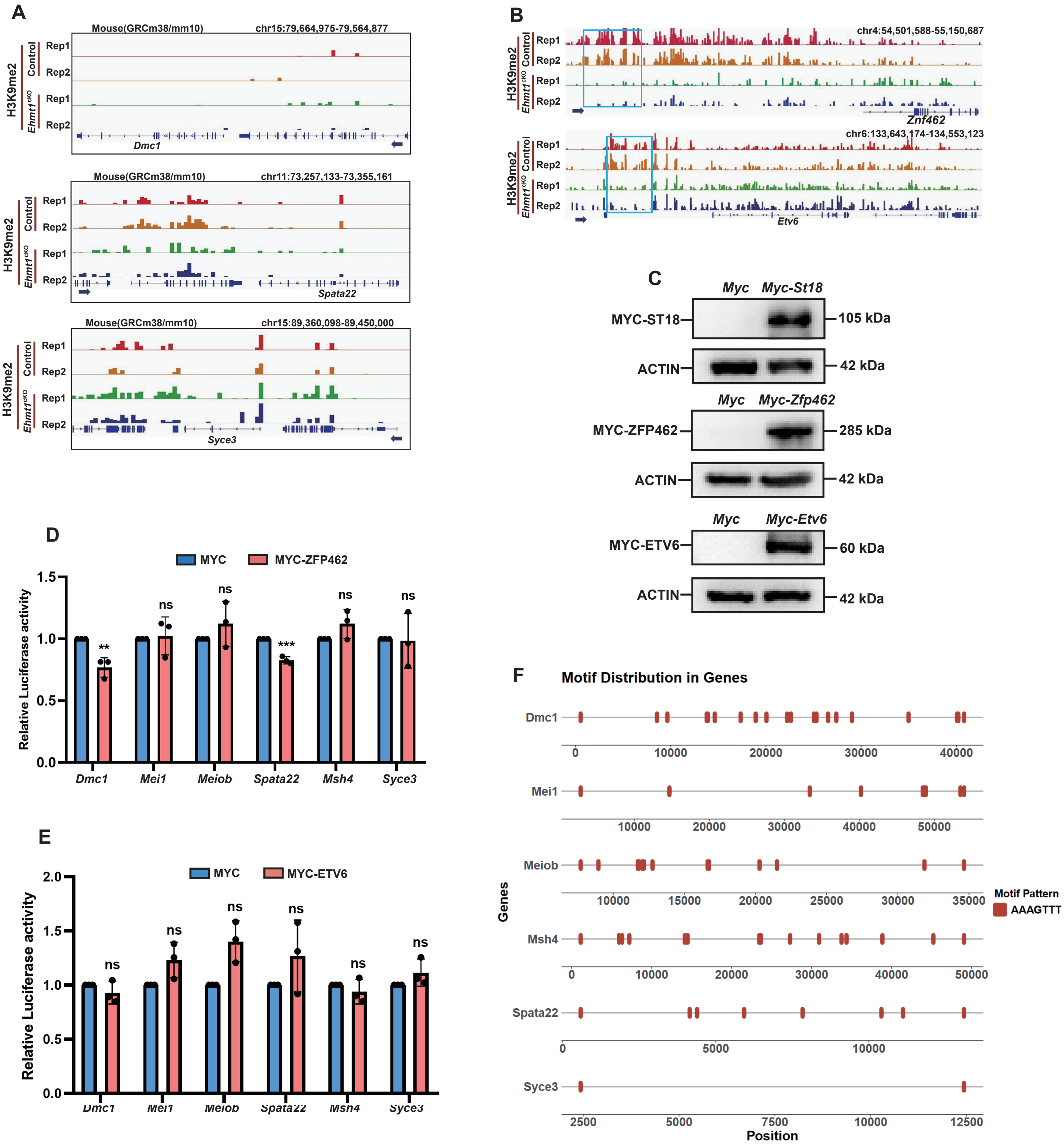

Supplementary Figure S6. ETV6 had no significant effect on transcription of meiotic genes. (**A**) Genome browser tracks depicting reads accumulation of H3K9me2 on meiotic genes *Dmc1, Spata22* and *Syce3* in control and *Ehmt1*^{cKO} SSEA1-positive cells using IGV software. (**B**) Genome browser tracks depicting reads accumulation of H3K9me2 on transcription factors *Etv6*, and *Zfp462* in Control and *Ehmt1*^{cKO} SSEA1-positive cells using IGV software. (**C**) Western blot analysis of MYC-ST18, MYC-ZFP462 and MYC-ETV6 in HEK293T cells that transfected with its overexpression plasmid. Actin levels were used as loading controls. Each experiment was performed at least three times. (**D**) Dual-luciferase reporter gene assays showed that when ZFP462 was overexpressed, the luciferase activity of the meiotic gene promoter regions in HEK293T cells was significantly decreased respectively. Data are expressed as mean ± SD. Two-tailed Student's t-test; $P > 0.05$ (ns), $P < 0.01$ (**). $P < 0.001$ (***). (**E**) Dual-luciferase reporter gene assays showed that when ETV6 were overexpressed, the luciferase activity of meiotic genes promoter regions in HEK293T cells was significantly decreased. Experiment was performed three times. Data are expressed as mean ± SD. Two-tailed Student's t-test; $P > 0.05$ (ns). (**F**) Distribution of the ST18 binding motif "AAAGTTT" within each of the 6 downregulated meiotic genes and their 1.0 kb upstream regulatory regions.

Supplementary Table 1

**Primer sequences are used in this study**		
**Target**	**Sequence (5' to 3')**	**Application**
Ehmt1-loxp	F: AGGAATTAGCTTTGAGAGTCGC	Genotyping
	R: CCTAACTCGTGAACCATAGGAA	
Tnap-Cre	F: CACGTCGATGGCCGCTCTA	
	R: TAAGGGCCAGCTCATTCCTCC	
Oct4-eGFP	F: TTCGAGGGCGACACCCTGGT	
	R: CTTCTCGTTGGGGTCTTTGCTC	
Gapdh	F: AGGTCGGTGTGAACGGATTTG	RT-qPCR
	R: TGTAGACCATGTAGTTGAGGTCA	
Dmc1	F: ATGAAGGAGGATCAAGTTGTGC	
	R: CATGCTTCTGCAACAGGTCAA	
Mei1	F: CAGGCTGTTCACAAGCTCAG	
	R: GAAGTGCTCTGTACTGCACG	
Meiob	F: CAGTCTCTCAGGAAGTATTGCTG	
	R: CCCCTTGCTCTGTGTGATAAAAT	
Spata22	F: TCTGACAGTTACGGTTCCCCT	
	R: GCTGAGGTCGAAAATCCCAAGT	
Msh4	F: CTGCGCGATTACAGCACTG	
	R: GTGGCTTCGAGCACTCCAA	
Msh5	F: CCGGAAACTACTCCTTCATCTCA	
	R: CCAATTCTTCTTCGACTCAGGAA	
Syce3	F: CTGATTCCGATCCTGGGGAAA	
	R: GGTTGCCTGCACTGAGATTTT	
Sycp1	F: CAAAAGCCCTTCACACTGTTCG	
	R: GTTTTCCCGACTGGACATTGTAA	
Prdm9	F: CTGAATACAAGTGGCTCAGAACA	
	R: CCTCATAGGCAAGGCCCTTTC	
Etv6	F: AGCAGGAACGAATTTCATACACG	
	R: GGCAGGTGGATCGAGTCTTC	
Klf12	F: CAGCGCCCTTGAGAACAGAAT	
	R: GTGGACGTTTGGAGACCCTTG	
St18	F: AGCAAAGGGGAACCTGAGTTT	
	R: GAACCTCGTTCAGCCTGTAGA	
Zeb1	F: CTGGCAAGACAACGTGAAAG	
	R: GCCTCAGGATAAATGACGGC	
Zfp462	F: AAGACTGCTCCTTTTACACCG	
	R: GGTCGATCATGTTGCGTTTGT	
Zfp608	F: AACAGAAGAAGGTGTCCTAGTGG	
	R: TTGTCGGTGATTCGCAAAACC	
Dmc1	F: GCACACAGTTGGGAATCAATGCC	Luciferase reporter systems
	R: ATGTAGCACAAAGGAGAGCGGA	

Mei1	F: GCAGTGGGTACCCAGAGGCAA	
	R: AAGGCAAAGTGTTGGAGGCTAG	
Meiob	F: GGATCAGAGGTTCAAGGTCAATTC	
	R: TCTCACGCACGTGTTCACGGT	
Spata22	F: CAGCCCTGCTCTGTGAACTTC	
	R: TACGTAGGGCAGGGGTGGTAATA	
Msh4	F: GGCATTGCTGGGTTGTCACTT	
	R: CTGGAAAGCGATGCAGCCTC	
Syce3	F: CAATCCAGTCATTGACTCCCAG	
	R: TACGTGCCTCGGGTAGAGCC	
St18-CDS Full length	F: ATGGATGCCGAGGTTGAAGAT	Overexpression plasmids construction
	R: CTACACATGGATGCCCTTCACC	
Etv6-CDS Full length	F: ATGTCTGAGACTCCTGCTCAGTC	
	R: GCTATTCCCGGGTCTCTTCCT	
Klf12-CDS Full length	F: ATGAATATCCATATGAAGAGGAAA	
	R: TCACACCAGCATGTGCCTCC	

Supplementary Table 2

**Antibodies used in this study**					
Antibodies	Species	Applications	Concentration	Sources	Cat #
DDX4/MVH	Rabbit	IF	1:300	Abcam	ab13840
DDX4/MVH	Mouse	IF	1:300	Abcam	Ab27591
H3K9Me2	Mouse	IF	1:200	Abcam	ab1220
H3K9Me2	Mouse	Cut&Tag	1:50	Abcam	ab1220
H3K27Me3	Rabbit	IF	1:200	Active Motif	39055
EHMT1	Mouse	IF	1:200	Abcam	ab41969
EHMT1	Mouse	WB	1:1000	Abcam	ab41969
SYCP3	Mouse	IF	1:100	Santa Cruz	sc-74569
SYCP3	Mouse	IF	1:200	Abcam	ab97672
SYCP3	Rabbit	IF	1:200	Novus	NB300-231
γH2AX	Rabbit	IF	1:200	CST	9718
DMC1	Rabbit	IF	1:100	Abcam	ab245217
DMC1	Rabbit	WB	1:800	Abcam	ab245217
RAD51	Rabbit	IF	1:200	Abcam	Ab133534
MLH1	Mouse	IF	1:50	Abcam	ab14206
SPATA22	Rabbit	WB	1:1000	Proteintech	16989-1-AP
MYC	Rabbit	WB	1:4000	Abclonal	AE070
β-ACTIN	Mouse	WB	1:2000	ZSGB-BIO	TA-09
HRP, Goat anti-mouse IgG	Goat	WB	1:4000	EASYBIO	BE0102
HRP, Goat anti-rabbit IgG	Goat	WB	1:4000	EASYBIO	BE0101
Dylight 488, Goat anti-mouse IgG	Goat	WB	1:500	Invitrogen	A32723
Dylight 488, Goat anti-rabbit IgG	Goat	WB	1:500	Invitrogen	A32731
Dylight 594, Goat anti- mouse IgG	Goat	WB	1:500	Invitrogen	A32742
Dylight 594, Goat anti-rabbit IgG	Goat	WB	1:500	Invitrogen	A32740
